# Calibrated Weighted Rank Aggregation for Virtual Screening Independently Rediscovers Privileged Vitamin D Receptor Ligand Scaffolds

**DOI:** 10.34133/csbj.0155

**Published:** 2026-07-13

**Authors:** Abylay Salimzhanov, Askar Boranbayev, Ferdinand Molnár, Siamac Fazli

**Affiliations:** ^1^ Department of Computer Science, School of Digital Sciences and Engineering, Nazarbayev University, Astana, Kazakhstan.; ^2^ Department of Biology, School of Sciences and Humanities, Nazarbayev University, Astana, Kazakhstan.

## Abstract

Computational drug discovery relies on virtual screening to prioritize a small number of compounds for experimental testing, yet combining heterogeneous *in silico* scoring methods into a single robust ranking remains challenging. We present Calibrated Weighted Rank Aggregation (CWRA), a data-driven rank-optimized score-fusion framework that (a) direction-corrects modality outputs, (b) harmonizes them via per-modality normalization, and (c) learns nonnegative fusion weights from known actives by optimizing a rank-based early-recognition objective (Boltzmann-enhanced discrimination of receiver operating characteristic [BEDROC]) under simplex constraints. Applied to the vitamin D receptor (VDR), we fuse machine-learning affinity predictors (GraphDTA endpoints, MLT-LE, TankBind, DrugBAN, and MolTrans), structure-based scores (AutoDock Vina and Boltz-2 affinity and confidence), and ligand similarity (Uni-Mol; computed in a split-honest manner by recomputing the active-set centroid on the training splits). Across 5 repeated random splits over the P=503 known VDR binders, evaluated on the drug-likeness prefiltered pool of $N^{\prime }=14,902$ compounds, CWRA achieves strong early enrichment on held-out actives (EF@1%=21.32±6.41, corresponding to 16 ± 5 recovered binders within the top 1% of the ranked list), substantially outperforming equal-weight fusion (EF@1%=10.79±2.81) and the strongest single modality (GraphDTA *IC*_50_; EF@1%=16.32±3.96). Performance remains strong at broader cutoffs (EF@2.5%=14.13±2.34, EF@5%=9.47±0.60, and EF@10%=7.13±0.72), indicating that BEDROC-optimized fusion of normalized scores improves both early prioritization and moderate-depth screening. To assess generalization beyond VDR, we further applied CWRA to the mechanistically distinct γ-aminobutyric acid type A (GABA_A_) receptor. Target-specific calibration again improved very early enrichment (EF@1%=25.41±3.96) over both equal-weight fusion (EF@1%=17.47±6.17) and the best individual modality at this cutoff (Boltz-2 confidence; EF@1%=11.64±2.12). Strict cross-target source-model transfer between VDR and GABA_A_ was weaker than target-specific recalibration, indicating that CWRA should be interpreted as a transferable target-adaptive framework rather than a universal fixed-weight model. A qualitative review of the top-ranked generated candidates suggests that CWRA preferentially selects chemically coherent VDR-like chemotypes, recapitulating established VDR pharmacophore patterns without imposing handcrafted structural rules.

## Introduction

Virtual drug screening (VS) has become a central component of early-stage drug discovery, enabling the identification of hit compounds for further optimization and experimental testing at a fraction of the cost of wet-lab high-throughput screening campaigns [1,2]. Modern VS pipelines span a wide spectrum of computational tools, from physics-based docking and structure-based scoring functions to ligand-based similarity searches and machine-learning (ML) models for activity or binding affinity prediction. Each of these modalities captures different and only partially overlapping aspects of protein–ligand recognition, so that the ranking of compounds can vary considerably across methods, especially in massive-scale screening and repurposing settings where many targets and ligand libraries are evaluated under heterogeneous predictors. Such efforts are supported by both experimental repositories (e.g., BindingDB [3]) and large-scale predicted or multitask resources [4,5].

A common strategy to exploit this diversity is to combine multiple predictors through consensus docking or consensus scoring, which has repeatedly been shown to improve VS performance over individual methods [6,7]. However, most existing consensus schemes either rely on unweighted score averaging or use heuristic and target-specific weight tuning. This makes it difficult to (a) transfer a given consensus strategy to new targets or new combinations of modalities and (b) quantify the statistical confidence that a ligand is consistently ranked near the top across methods.

Here, we address this gap by formulating multimodal VS as a data-driven fusion problem. Methodologically, our work builds on the statistical framework of Calibrated Weighted Rank Aggregation (CWRA) but extends it in several ways for the context of multimodal VS. First, we propose a target-specific calibration scheme that derives modality weights directly from known active binders by optimizing an early-recognition objective (Boltzmann-enhanced discrimination of receiver operating characteristic [BEDROC]) and related early-enrichment behavior, rather than relying on heuristic or manually tuned weights. Second, we integrate these calibrated weights into a unified consensus score that combines 11 heterogeneous computational modalities after direction correction and normalization, spanning ML affinity predictors, molecular docking, structure-based predictors, and ligand similarity. Third, we apply this consensus scoring to a large dataset of *de novo* generated molecules, using CWRA to prioritize candidates for expert review and experimental follow-up.

We applied CWRA to the vitamin D receptor (VDR) as the primary case study, aggregating 11 complementary computational modalities: graph-neural-network-based drug–target affinity (DTA) models, including GraphDTA [8], multitask learning affinity predictors (MLT-LE) [9], Boltz-2-based affinity predictions [10], classical molecular docking with AutoDock Vina [11], Uni-Mol similarity [12], TankBind affinity [13], DrugBAN affinity [14], and MolTrans affinity [15]. VDR was selected because its ligand chemistry and structure–activity relationships are extensively characterized, allowing both quantitative evaluation and domain-informed interpretation of the prioritized compounds. To assess whether the ranking framework was specific to the VDR dataset, we additionally applied the same computational pipeline to the γ-aminobutyric acid type A (GABA_A_) receptor, an ionotropic ligand-gated ion channel that is pharmacologically and mechanistically distinct from VDR. These secondary analyses were intended as cross-target tests of methodological portability rather than as detailed target-specific medicinal chemistry studies. We therefore retained VDR as the principal target for the generative, structural, and medicinal chemistry analyses. Before applying CWRA, we fine-tune several deep generative models on known VDR binders to generate a set of novel VDR binding candidates. Our results show that a simple, well-calibrated, data-driven weighting of modalities substantially improves early enrichment over any individual predictor or naïve averaging and provides a general recipe for integrating heterogeneous computational evidence in VS campaigns. Notably, CWRA’s top-ranked generated compounds independently converge on secosteroid A-ring C2α-modifications and lactone side-chain substitutions, privileged structural motifs validated across decades of VDR medicinal chemistry [16,17], demonstrating that BEDROC-optimized fusion of normalized scores can capture established structure–activity principles without explicit chemical knowledge.

## Methods

### Reference VDR binder dataset

VDR is a nuclear receptor transcription factor with well-established roles in calcium/phosphate homeostasis and immune modulation [18–20]. We constructed a reference set of reported VDR binders by aggregating compounds annotated with VDR activity from ChEMBL, PubChem, and ZINC [21–23]. All structures were represented as Simplified Molecular-Input Line-Entry System (SMILES) strings and were required to pass RDKit parsing/validity checks [24]. We standardized SMILES by removing stereochemical/isomeric annotations, generating RDKit-canonical SMILES, and removing exact duplicates across sources, yielding P=503 unique reference compounds (labeled *positives*). Throughout, we use the term *VDR binders* to denote compounds reported to interact with VDR irrespective of binding site, since some ligands may engage noncanonical regions in addition to the classical ligand-binding pocket (LBP).

### *De novo* candidate generation and library assembly

To expand chemical space beyond known binders, we generated *de novo* candidates using 3 SMILES-based generative models with complementary inductive biases: REINVENT, GMDLDR, and Transmol [25–27]. This follows the broader use of ML-generated virtual chemical spaces for downstream search and prioritization [28].

Each generator was initialized from a pretrained checkpoint and adapted to the VDR ligand distribution via transfer learning on the standardized reference set (see Section S1.2 of the Supplementary Materials for training details and evaluation).

All generated SMILES were postprocessed with a uniform RDKit pipeline: invalid SMILES were discarded, valid molecules were canonicalized (including removal of stereochemical annotations for consistency with the reference standardization), and exact duplicates were removed. To prevent trivial memorization of fine-tuning data, any generated molecule whose canonical SMILES matched a molecule present in the corresponding fine-tuning/training subset was removed. Candidates were then pooled per generator and merged across generators to form a nonredundant screening library used in downstream multimodal scoring.

Because the generative models used in this study output nonstereospecific SMILES representations, generated candidates were standardized, deduplicated, and tracked at the level of constitutional molecular graphs. Stereochemical annotations were therefore not used as constraints during the primary ranking of generated molecules. For structure-based scoring, 3-dimensional (3D) ligand conformations or protein–ligand complexes were generated from these standardized molecular graphs prior to docking or complex prediction. However, possible stereoisomers were not systematically enumerated. Consequently, AutoDock Vina and Boltz-2 scores for generated candidates should be interpreted as structure-based assessments of plausible 3D realizations of the corresponding molecular graph rather than as fully stereochemistry-resolved evaluations of a unique biologically preferred stereoisomer. Since the same standardization protocol was applied consistently across the reference and generated libraries, this limitation primarily affects the interpretation of individual top-ranked candidates as experimentally actionable structures rather than the comparative CWRA enrichment analysis.

For descriptive analyses, we additionally grouped generated molecules by cross-generator overlap on canonical SMILES: molecules produced by exactly 1 generator (G1), by exactly 2 generators (G2), and by 3 generators (G3).

### Bemis–Murcko scaffold analysis

Bemis–Murcko molecular scaffolds [29] were computed using RDKit [24] to assess the scaffold-level novelty of the generated library. Scaffold extraction was performed after the same molecular standardization procedure used for CWRA scoring. For each molecule, the Bemis–Murcko scaffold was generated from the standardized molecular graph and converted to canonical SMILES. Unique scaffolds were then counted separately for the 503 reference actives, the drug-likeness-filtered generated library, and the CWRA top 100 short list. A generated scaffold was considered reference-novel if its canonical Bemis–Murcko scaffold SMILES was absent from the scaffold set derived from the 503 reference actives.

### Candidate pool, modality table, and molecular descriptors

We assembled a unified screening table comprising (a) the P=503 known VDR binders (positives) and (b) a substantially larger set of unlabeled *de novo* candidates generated by the 3 models (see the “*De novo* candidate generation and library assembly” section). Each row corresponds to one molecule and contains a source identifier, canonical SMILES, the positive label where available, outputs from the M=11 computational scoring modalities used for fusion (see the “Computational scoring modalities” section), and a set of standard 2-dimensional (2D) physicochemical descriptors computed with RDKit from the canonical SMILES (e.g., molecular weight [MW], cLogP, topological polar surface area [tPSA], number of hydrogen-bond donors [HBD]/number of hydrogen-bond acceptors [HBA], number of rotatable bonds, ring counts, FractionCSP3, heavy-atom count, formal charge, molar refractivity [MR], stereocenter count, BertzCT, LabuteASA, quantitative estimate of drug-likeness [QED], and SAScore). After excluding designated sources to prevent leakage, the working pool contained N=16,196 molecules.

Unless explicitly stated otherwise, these RDKit descriptors are used for descriptive analyses and optional filtering/diagnostics (e.g., coarse drug-likeness screening) and are not used in the CWRA weight-learning objective itself, which is defined purely over the modality-derived scores.

#### Drug-likeness prefilter

Before learning CWRA fusion weights, we applied a simple drug-likeness prefilter to unlabeled candidates to remove extreme outliers with MW > 700 Da or rotatable bond count RotB > 15. Known actives were never removed (all P=503 reference binders were retained; MW: max 677.47, mean 464.57 ± 66.21; RotB: max 18, mean 8.52 ± 3.13). A small fraction of known binders exceed the RotB threshold (9/503), but the filter is applied only to unlabeled candidates to focus follow-up on an experimentally tractable region rather than to assert nonbinders. In total, the prefilter excluded 1,294 unlabeled compounds (106 by MW only, 903 by RotB only, 283 by both criteria, and 2 due to SMILES parsing failures), reducing the pool from N=16,196 to $N^{\prime }=14,902$ for CWRA optimization. This step serves 2 purposes: (a) very large poly(ethylene glycol)-linked molecules and bifunctional degraders such as proteolysis-targeting chimeras may score well on binding-competent fragments while being outside the intended chemical space of classical VDR modulation and (b) removing such outliers prevents extreme modality values from compressing the dynamic range of score normalization and subsequent ranking.

### Computational scoring modalities

Each molecule was evaluated with *M* = 11 complementary scoring modalities spanning 3 categories: (a) deep-learning predictors, (b) structure-based scoring, and (c) ligand-based similarity.

#### Deep-learning predictors

GraphDTA [8] produces 3 predicted affinity endpoints (${\text{pK}}_{d}$, ${\text{pK}}_{i}$, and ${\mathrm{pIC}}_{50}$) from ligand and protein representations. MLT-LE [9] provides an additional predicted ${\mathrm{pK}}_{d}$ score. We also include 2 drug–target interaction predictors: DrugBAN [14] and MolTrans [15], which model interaction patterns via bilinear attention and transformer-based architectures, respectively.

#### Structure-based scoring

AutoDock Vina docking against the VDR ligand-binding domain (Protein Data Bank [PDB] ID: 1DB1) [11,30] yields physics-based docking energies. Boltz-2 [10] contributes 2 outputs: a predicted binding score and a confidence estimate of the predicted complex structure. TankBind [13] predicts a binding site/pose and provides an affinity-like score; we use the resulting scalar score as an additional structure-based modality.

#### Ligand-based similarity

Uni-Mol [12] provides a learned molecular similarity score computed in Uni-Mol embedding space (cosine similarity to a reference embedding derived from the known VDR binders). This modality captures ligand-space proximity to the reference active set independently of target structure information. During each repeated train/test split, the reference centroid was recomputed using only the training-split actives.

For modalities where lower raw values indicate stronger binding evidence (e.g., Vina and the Boltz-2 affinity score), we inverted the direction so that larger values consistently indicate stronger binding evidence. Missing modality outputs were mean-imputed per modality prior to normalization.

### CWRA meta-score for multimodal ranking

Let $x_{i,m}$ denote the raw score of molecule *i* under modality $m\in \left\{1,...,\;M\right\}$ with M=11. Missing modality outputs are mean-imputed per modality. Each modality is then normalized to the unit interval to produce $z_{i,m}\in \left[0,1\right]$ using min–max normalization:

$$z_{i,m}=\frac{x_{i,m}-{\text{min}}_{j}x_{j,m}}{{\text{max}}_{j}x_{j,m}-{\text{min}}_{j}x_{j,m}},$$ (1)

where the extrema are taken over the $N^{\prime }$ compounds in the prefiltered pool used for CWRA training. For modalities with a lower-is-better direction, we apply $z_{i,m}\leftarrow 1-z_{i,m}$ so that larger values consistently indicate stronger binding evidence.

#### Fused meta-score

We define the CWRA fused score as a convex combination of normalized modality scores:

$$s_{i}=\sum_{m=1}^{M}w_{m}z_{i,m},\;\sum_{m=1}^{M}w_{m}=1,\;w_{m}\in \left[w_{\min},w_{\text{max}}\right],$$ (2)

with $w_{\text{min}}=0.03$ and $w_{\text{max}}=0.25$. The simplex constraint and per-modality bounds are enforced by projecting candidate weight vectors onto the capped simplex $\begin{array}{c} \left\{w:\sum_{m}w_{m}=1,w_{\text{min}}\leq w_{m}\leq w_{\text{max}}\right\} \end{array}$ via bisection on the Lagrange multiplier [31]. The lower bound prevents complete removal of any modality, whereas the upper bound preserves the intended interpretation of CWRA as an interpretable multimodal consensus model rather than an automatic single-modality selector.

#### BEDROC objective

We optimize weights for early recognition using the BEDROC metric [32], which assigns each active at a fractional rank *r* an exponentially decaying reward $e^{-\alpha r}$. We use α=100, corresponding to emphasis on approximately the top 1% of the ranked list (about the top ~149 compounds in our $N^{\prime }=\text{14,902}$ pool). Compared to enrichment-factor objectives at hard cutoffs, BEDROC provides a more graded early-enrichment signal that better rewards incremental improvements near the top of the list.

#### Optimization

Weights are optimized using differential evolution (DE) [33] with the best1bin strategy, up to 1,000 iterations and a population size of 15 × *M* (SciPy default). The “fair” variant constrains DE to $\left[w_{\min},w_{\text{max}}\right]$ per modality, and each candidate vector is projected onto the capped simplex before objective evaluation. The DE seed is fixed at 42 for reproducibility.

#### Repeated splits on actives

To estimate generalization to unseen actives, we perform 5 repeated random splits over the P=503 known actives, using a fixed training fraction of 0.85 (seed = 42). In each repeat, weights are optimized using only the training-split actives and enrichment is measured on the held-out actives, while all nonactive compounds remain in the ranking pool; evaluation always ranks against the same full pool of $N^{\prime }$ compounds. We report the mean ± standard deviation (SD) of enrichment factors (EFs), area under the receiver operating characteristic curve (AUROC), area under the precision–recall curve (AUPRC), and BEDROC across the 5 repeats.

### Positive–unlabeled conformal selection pipeline

Because only positives are confirmed and all other molecules are unlabeled, we operate in a positive–unlabeled (PU) setting. We implement a 5-step pipeline (A to E) to (a) construct diagnostic negative subsets, (b) compute a fused ranking score, (c) calibrate conformal *P* values against an approximate null, and (d) select a short list from the unlabeled pool.

#### Step A: Reliable negatives for diagnostics

To obtain a conservative negative subset for diagnostics, we construct reliable negatives (RNs) from the unlabeled pool using 2 heuristics applied across all *M* = 11 modalities:

1. Low-score heuristic: we compute a baseline score as the mean of the direction-corrected min–max normalized modality values $z_{i,m}$ and restrict candidates to the bottom 20% (bottom_q = 0.2) among unlabeled molecules.
2. Dissimilarity-to-actives heuristic: we remove candidates whose maximum Tanimoto similarity to any known active exceeds 0.35, computed using RDKit Morgan fingerprints (radius 2, 2,048 bits).

From the resulting candidates, we retain 2,699 RNs in the current run (the similarity filter reduced the RN pool below the bottom-quantile target). This step assigns PU labels: positives (+1), RNs (0), and remaining unlabeled (−1).

##### Drug-likeness prefilter (pipeline stage)

After RN assignment, we apply the same coarse drug-likeness filter as in the “Candidate pool, modality table, and molecular descriptors” section to unlabeled molecules within the pipeline. Unlabeled compounds exceeding MW > 700 Da or RotB > 15 are relabeled as excluded (−2) and removed from downstream selection. In the current run, this excluded 1,122 unlabeled molecules, leaving 11,872 unlabeled candidates. Positives and RNs are never affected by this filter.

#### Step B: CWRA scoring with pre-computed weights

We compute normalized modality scores $z_{i,m}$ and the CWRA meta-score $s_{i}$ (Eq. (2)) using the same min–max normalization and direction harmonization described above. Rather than reoptimizing weights within the pipeline, we apply the mean weights obtained from the repeated split evaluation (see the “CWRA meta-score for multimodal ranking” section). This produces a single fused score $s_{i}$ for each molecule, which is used for downstream conformal calibration and selection.

#### Step B2: Calibration-negative construction (null set)

Conformal calibration requires a reference set that approximates the null (nonbinder) score distribution. We use the RNs constructed in step A as calibration negatives, and compute their CWRA meta-scores to form the calibration score set $\left\{s_{j}^{\mathrm{cal}}\right\}$. Calibration negatives are excluded from the final selection universe by design, since selection is performed over the remaining unlabeled pool.

#### Step C: Unweighted conformal *P* values

Let ${\left\{s_{j}^{\mathrm{cal}}\right\}}_{j=1}^{n_{\mathrm{cal}}}$ denote the CWRA meta-scores of the calibration negatives (here, the RNs from step A). For each molecule *i*, we compute a one-sided conformal *P* value:

$$P_{i}=\frac{1+\sum_{j=1}^{n_{\text{cal}}}I\left[s_{j}^{\text{cal}}\geq s_{i}\right]}{n_{\mathrm{cal}}+1}.$$ (3)

A small $P_{i}$ indicates that $s_{i}$ exceeds most calibration negatives (i.e., is atypically large under the approximate null). The minimum achievable *P* value under Eq. (3) is $P_{\text{min}}=1/\left(n_{\text{cal}}+1\right)$.

#### Step D: Weighted conformal *P* values under covariate shift

If the calibration-negative distribution differs from the target unlabeled distribution (covariate shift), we additionally compute weighted conformal *P* values as a robustness check. We construct a feature vector by concatenating the *M* = 11 modality values with 17 RDKit descriptors (MW, cLogP, tPSA, HBD, HBA, RotB, RingCount, AromaticRingCount, FractionCSP3, HeavyAtomCount, FormalCharge, MR, NumStereocenters, BertzCT, LabuteASA, QED, and SAScore). A probabilistic domain classifier is trained to discriminate calibration negatives from the target unlabeled pool, and its predicted probabilities are converted into importance weights $\omega_{j}$, which are clipped to a bounded range to avoid extreme reweighting (here, $\omega_{j}\in \left[1/\text{clip},\;\text{clip}\right]$, with clip = 20, i.e., [0.05, 20]).

Weighted conformal *P* values are then computed as

$$P_{i}^{\left(w\right)}=\frac{1+\sum_{j=1}^{n_{\text{cal}}}\omega_{j}I\left[s_{j}^{\text{cal}}\geq s_{i}\right]}{1+\sum_{j=1}^{n_{\mathrm{cal}}}\omega_{j}}.$$ (4)

As a diagnostic of weight concentration, we compute the effective sample size:

$$n_{\text{eff}}=\frac{{\left(\sum_{j}\omega_{j}\right)}^{2}}{\sum_{j}\omega_{j}^{2}},$$ (5)

where a smaller $n_{\text{eff}}$ indicates stronger concentration of importance weights. For the final selection step, we use the unweighted *P* values (Eq. (3)).

#### Step E: Final short list and conformal annotation

For downstream expert review, we select a short list from the remaining unlabeled pool using the CWRA meta-score and conformal *P* values as significance annotations. After excluding (a) known actives, (b) calibration negatives (RN), and (c) unlabeled candidates removed by the drug-likeness prefilter, we rank the remaining unlabeled candidates by $s_{i}$ and define a selection universe as the top B molecules by $s_{i}$ (here, B=1,000). Within this universe, we apply Benjamini–Hochberg multiple-testing correction to the conformal *P* values with target false discovery rate (FDR) $q_{0}=0.05$ and retain candidates whose adjusted *q* values fall below the corresponding threshold (for visualization, we annotate * for q<0.05, ** for q<0.01, and *** for q<0.001). Selected molecules are reported with their CWRA meta-scores, per-modality normalized values, and conformal *P* values to support follow-up and expert inspection.

### Structural feature enrichment statistics

For each compound group, we compute the prevalence of predefined structural features using RDKit-based feature annotations. To assess enrichment or depletion between groups, we construct 2 × 2 contingency tables (feature present/absent × group) and apply 2-sided Fisher’s exact tests, reporting odds ratios and *P* values. We apply a Benjamini–Hochberg FDR correction across the full set of Fisher tests shown in the panel (24 tests total) and annotate significance using adjusted *q* values (*q<0.05, **q<0.01, and ***q<0.001).

### Expert review and visualization

To facilitate chemical plausibility checks, we generate 2D depictions of the top-ranked short list with per-molecule annotations including MW, cLogP, tPSA, HBD, HBA, and RotB computed on the fly from SMILES via RDKit. These panels are shared with domain experts for visual inspection to flag obvious artifacts (e.g., reactive motifs, implausible chemotypes, or excessive lipophilicity). This review step is treated as a postselection quality-control step, separate from the statistical calibration procedure.

### Post hoc interaction analysis of Boltz-2 affinity predictions with PLIP

To characterize protein–ligand interactions beyond affinity predictions, we performed post hoc interaction analysis using Protein–Ligand Interaction Profiler (PLIP) [34]. PLIP interaction analysis was performed using the default geometric criteria implemented in the PLIP web server configuration, including hydrogen bonds (donor–acceptor distance ≤ 4.1 Å and D–H–A angle ≥ 100°), hydrophobic contacts (distance between hydrophobic atom centers ≤ 4.0 Å), π-stacking interactions (ring-centroid distance ≤ 5.5 Å, angular deviation ≤ 30°, and ring offset ≤ 2.0 Å), and salt bridges (charge-center distance ≤ 5.5 Å). No custom interaction thresholds or postprocessing filters were applied. The analysis was limited to a small set of representative structures: 3 calcitriol references and 5 top-ranked generated compounds.

PLIP analysis was applied to Boltz-2 affinity-predicted structures for each ligand. For calcitriol, 3 reference structures were analyzed: a low-resolution crystallographic structure (PDB ID: 1DB1), a high-resolution crystallographic structure (PDB ID: 7QPP), and a Boltz-2 affinity-predicted structure with calcitriol as the ligand. Generated compounds were analyzed using their corresponding Boltz-2 affinity-predicted structures in complex with VDR.

Hydrogen-bond and hydrophobic interactions were extracted from PLIP output and categorized using a residue-tiering scheme reflecting established structure–function relationships in the VDR LBP. Residues were assigned to Tier1, Tier2, or Tier3 based on published mutagenesis, alanine-scanning, and structure–activity relationship studies of calcitriol binding. In particular, residues involved in canonical calcitriol anchoring, including Y143, S237, R274, S278, H305, and H397, were classified as Tier1 hydrogen-bond residues, reflecting their critical contribution to high-affinity agonist binding [19,35]. Residues contributing secondary or context-dependent interactions were assigned to Tier2, while peripheral or nonconserved contacts were assigned to Tier3.

Hydrophobic interactions were categorized analogously based on their proximity to the secosteroid core and aliphatic side chain of calcitriol, as established from crystallographic analyses of the VDR ligand-binding domain. Interaction scores and tier-weighted summaries were used exclusively for descriptive and comparative analyses and were not used for ranking or scoring within the CWRA framework.

## Results

### Generative model output

#### Generative overlap with the reference set

Across all 3 generators, direct recreation of held-out reference ligands was limited under our sampling and filtering settings, with recreation coverage ranging from 1.6% to 6.8% of the 503-ligand reference set (Table S2). This indicates that the downstream screening and scaffold rediscovery results are not driven primarily by exact duplication of known actives. Because recreation rate measures overlap rather than binding strength, we evaluate generated candidates using orthogonal computational predictors and prioritize compounds using the proposed multimodal fusion framework.

### Overlap of generative models

**Fig. 1.**
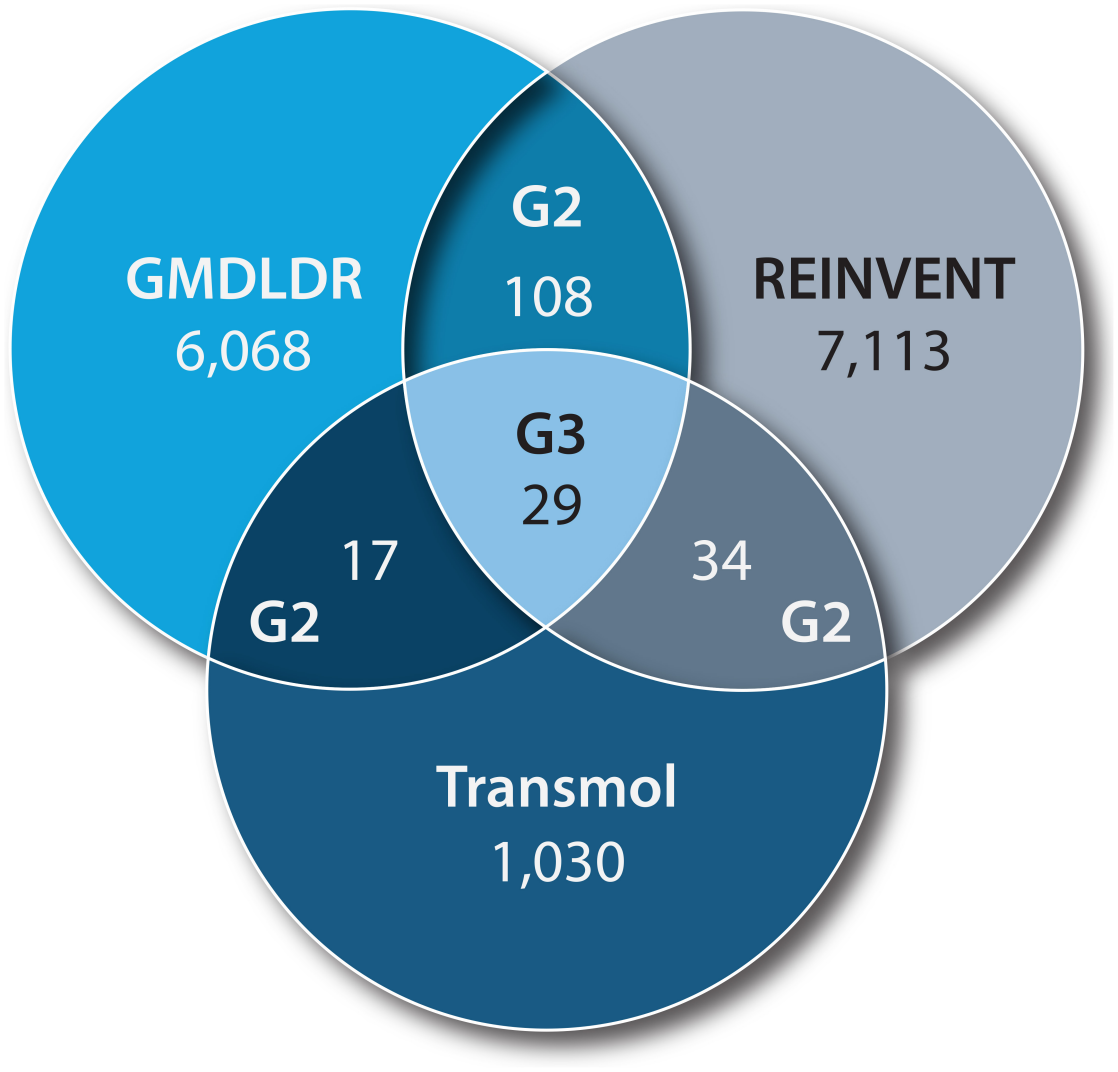
Overlap of generated vitamin D receptor (VDR) candidate libraries across the 3 generative models. Numbers indicate the size of unique and shared subsets.

Figure 1 shows the overlap of the 3 generated libraries after deduplication by canonical SMILES. The libraries are largely distinct: most molecules are unique to a single generator, and intersections are small. For example, only 29 molecules are shared by 3 generators, indicating a limited but nonzero consensus region across architectures.

### Scaffold-level novelty of the generated library

To assess whether the generated candidates merely reproduced known VDR chemotypes or expanded the chemical space beyond the reference binders, we computed Bemis–Murcko molecular scaffolds [29] for all generated candidates and for the 503 reference binders using RDKit [24] (see Table 1). The reference binder set contained 85 unique Bemis–Murcko scaffolds, while the drug-likeness-filtered generated sublibrary (14,399 compounds) contained 6,770 unique scaffolds, of which 6,691 (98.8%) were absent from the reference scaffold set, indicating broad scaffold-level diversity and arguing against simple scaffold-level memorization. We denote by G*k* the set of compounds independently produced by exactly *k* of the 3 architecturally distinct generators (REINVENT, Transmol, and GMDLDR). Stratifying the generated library by cross-generator consensus revealed a strong monotonic trend: the single-generator output (G1) was almost entirely scaffold-novel relative to the reference set (98.8%), the 2-generator class (G2) was intermediate (40% novel), and compounds on which all 3 generators independently converged (G3) were almost exclusively reference-like (0% novel). Because the 3 architectures share no parameters and were trained independently, this monotonic convergence toward known chemotypes at higher consensus levels is unlikely to reflect single-model memorization and is consistent with the 3 generators each recovering the same underlying VDR pharmacophore signal.

**Table 1.** Bemis–Murcko scaffold novelty analysis, stratified by cross-generator consensus. G*k* denotes compounds independently produced by exactly *k* of the 3 architecturally independent generators (REINVENT, Transmol, and GMDLDR). The generated library is broadly scaffold-novel relative to the 503 reference actives (98.8% overall), with the single-generator output (G1) almost entirely novel and the 3-generator-consensus output (G3) almost entirely reference-like. The CWRA top 100 inherits the consensus-driven concentration on reference-like scaffolds, consistent with BEDROC-calibrated recovery of known VDR pharmacophore types.

Subset	Compounds	Unique scaffolds	Novel *vs.* reference
Reference actives	503	85	—
Generated, G1	14,211	6,770	98.8% (6,691/6,770)
Generated, G2	159	20	40% (8/20)
Generated, G3	29	6	0% (0/6)
All generated, G1 to G3	14,399	6,770	98.8% (6,691/6,770)
CWRA top 100	100	36	5.6% (2/36)

CWRA, Calibrated Weighted Rank Aggregation; BEDROC, Boltzmann-enhanced discrimination of receiver operating characteristic; VDR, vitamin D receptor

The CWRA top 100 short list contained 36 unique Bemis–Murcko scaffolds, of which 34 overlapped with the reference scaffold set and 2 were novel (5.6%). The top 100 scaffold profile therefore resembles the G3 consensus class rather than the G1 single-generator class, reflecting the combined effect of cross-generator consensus, which already filters toward reference-like chemistry before scoring, and BEDROC-calibrated prioritization, which further sharpens this preference toward VDR-active chemotypes. The generated library thus provides broad scaffold exploration, whereas CWRA acts as a biologically calibrated prioritization layer within this diverse chemical space.

### Cross-referencing individual modalities

**Fig. 2.**
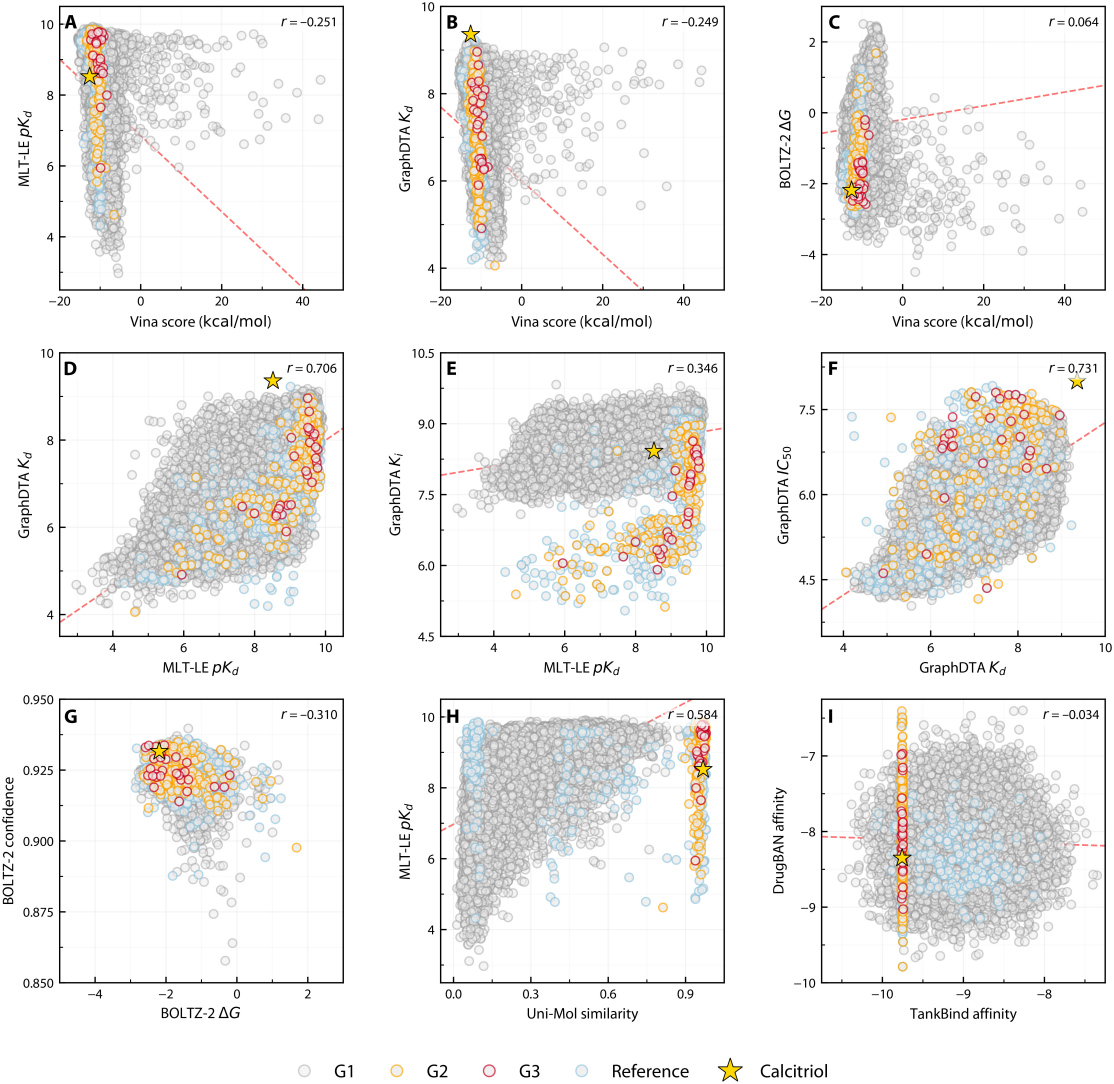
Representative pairwise relationships between unimodal scoring functions. Scatterplots show selected pairwise comparisons between modalities used in Calibrated Weighted Rank Aggregation (CWRA). Each point represents a screened compound, colored by generative model overlap group (G1, single generator; G2, 2 generators; G3, 3 generators); reference compounds are marked distinctly, and calcitriol is highlighted with a yellow star. Dashed red lines indicate least-squares linear fits, and inset values report Pearson correlation coefficients (*r*). (A) MLT-LE *pK_d_ vs.* AutoDock Vina score. (B) GraphDTA *K_d_ vs.* AutoDock Vina score. (C) Boltz-2 Δ*G vs.* AutoDock Vina score. (D) GraphDTA *K_d_ vs.* MLT-LE *pK_d_*. (E) GraphDTA *K_i_ vs.* MLT-LE *pK_d_*. (F) GraphDTA *IC*_50_ *vs.* GraphDTA *K_d_*. (G) Boltz-2 confidence *vs.* Boltz-2 Δ*G*. (H) MLT-LE *pK_d_ vs.* Uni-Mol similarity. (I) DrugBAN affinity *vs.* TankBind affinity. The panels illustrate internal consistency within related model families, moderate agreement between some predictors, and substantial complementarity across others, motivating multimodal normalized-score fusion.

**Fig. 3.**
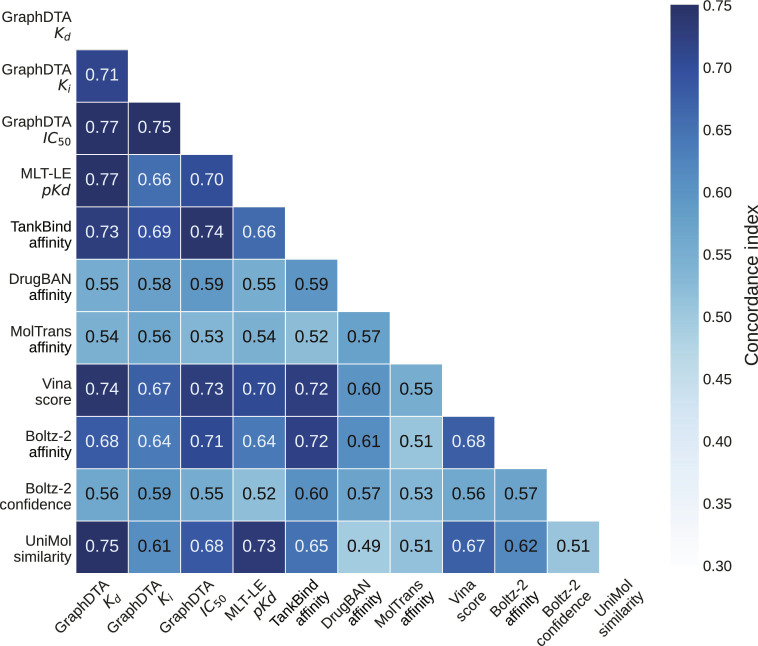
Concordance between binding modalities. Pairwise rank concordance across all modalities, reported as $C=\left(\tau_{b}+1\right)/2$, where $\tau_{b}$ is Kendall’s τ-b computed on direction-corrected raw scores (pairwise complete observations). Values near 0.5 indicate little rank association, values above 0.5 indicate partial agreement, and values below 0.5 indicate inverse ranking.

To examine how strongly the individual modalities agree (or disagree) on the same candidate set, we inspected representative pairwise scatterplots (Fig. 2) and summarized overall pairwise rank concordance across modalities (Fig. 3). We consider physics-based docking (AutoDock Vina), deep-learning affinity predictors (MLT-LE and GraphDTA endpoints), Boltz-2 affinity, ligand similarity (Uni-Mol), and deep-learning DTA models with distinct inductive biases (TankBind, DrugBAN, and MolTrans).

#### Pairwise relationships

Representative pairwise comparisons between modalities are shown in Fig. 2A to I. Overall, the plots reveal 3 consistent patterns: (a) classical docking (Vina) is only weakly aligned with affinity predictors, (b) affinity predictors show strong within-family agreement, and (c) several newer DTA models provide largely nonredundant signals, supporting multimodal fusion.

Docking shows weak to moderate negative linear association with learned affinity predictors (Fig. 2A: Vina *vs.* MLT-LE ${\text{pK}}_{d}$, r=−0.251; Fig. 2B: Vina *vs.* GraphDTA $K_{d}$, r=−0.249). The negative sign is expected because more favorable docking corresponds to more negative Vina scores, whereas higher values of ML endpoints indicate stronger predicted binding. Vina also exhibits only weak association with Boltz-2 ΔG (Fig. 2C: Vina *vs.* Boltz-2 ΔG, r=0.064), indicating limited concordance between physics-based docking and structure-based deep-learning scoring.

In contrast, strong internal consistency is observed among the ML affinity predictors. MLT-LE agrees strongly with GraphDTA $K_{d}$ (Fig. 2D: MLT-LE ${\text{pK}}_{d}$ *vs.* GraphDTA $K_{d}$, r=0.706) and shows moderate association with GraphDTA $K_{i}$ (Fig. 2E: MLT-LE ${\text{pK}}_{d}$ *vs.* GraphDTA $K_{i}$, r=0.346). Within the GraphDTA family, predicted endpoints are strongly concordant (Fig. 2F: GraphDTA $K_{d}$ *vs.* GraphDTA ${\mathrm{IC}}_{50}$, r=0.731), consistent with shared architecture and training objective family while still leaving room for endpoint-specific differences.

Boltz-2 confidence behaves differently from affinity estimates: it shows a moderate inverse relationship with Boltz-2 ΔG (Fig. 2G: Boltz-2 ΔG *vs.* Boltz-2 confidence, r=−0.310), suggesting that higher-confidence structural predictions are not necessarily associated with more favorable predicted binding energies. Ligand-based similarity partially overlaps with affinity prediction: Uni-Mol similarity is moderately correlated with MLT-LE (Fig. 2H: MLT-LE ${\mathrm{pK}}_{d}$ *vs.* Uni-Mol similarity, r=0.584), consistent with the idea that proximity to known ligands captures some, but not all, of the information used by learned affinity predictors.

Finally, the newer DTA models can be weakly related or effectively orthogonal, indicating complementarity. For example, TankBind and DrugBAN show near-zero correlation (Fig. 2I: TankBind *vs.* DrugBAN, r=−0.034), implying that these models emphasize different inductive biases (e.g., geometric pocket reasoning versus sequence/attention-driven interaction modeling). Together, these panel-level relationships support the motivation for CWRA: even when some modalities are correlated (Fig. 2D to F), others contribute distinct ordering signals (Fig. 2A to C and I), and fusion is therefore expected to improve ranking robustness.

Across panels, calcitriol (yellow star) consistently appears in regions associated with strong predicted binding and/or high similarity, providing an internal sanity check that a canonical high-affinity ligand receives broad support across heterogeneous predictors.

#### Global rank concordance

Overall rank agreement between modalities is summarized in Fig. 3, which reports pairwise concordance indices across all M=11 modalities (including Boltz-2 confidence). Concordance is computed as $C=\left(\tau_{b}+1\right)/2$, where $\tau_{b}$ is Kendall’s τ-b on direction-corrected raw scores (lower-is-better modalities are sign-flipped so that higher indicates stronger binding evidence), using pairwise complete observations. Across modality pairs, concordance ranges from 0.49 to 0.77, indicating substantial shared signal but also meaningful heterogeneity.

The strongest agreement occurs within the GraphDTA endpoints ($K_{d}$–$K_{i}$: 0.71; $K_{d}$–${\mathrm{IC}}_{50}$: 0.77; $K_{i}$–${\mathrm{IC}}_{50}$: 0.75), consistent with shared model architecture. MLT-LE shows high concordance with GraphDTA, particularly with GraphDTA $K_{d}$ (0.77) and moderate agreement with the other endpoints (0.66 to 0.70). Structure-based scores align with this affinity-prediction “core”: Vina is strongly concordant with GraphDTA $K_{d}$ and GraphDTA ${\mathrm{IC}}_{50}$ (0.74 and 0.73) and with MLT-LE (0.70). TankBind, while derived from geometric modeling, also exhibits substantial rank agreement with several modalities (e.g., with GraphDTA ${\mathrm{IC}}_{50}$, 0.74; with Vina, 0.72; and with Boltz-2 affinity, 0.72), suggesting partial redundancy in the high-scoring region despite differing inductive biases. Boltz-2 affinity shows moderate to strong concordance with the affinity predictors and docking (e.g., with GraphDTA ${\mathrm{IC}}_{50}$, 0.71; with MLT-LE, 0.64; and with Vina, 0.68).

Ligand-based Uni-Mol similarity is strongly concordant with GraphDTA $K_{d}$ (0.75) and MLT-LE (0.73) and moderately with Vina (0.67) and Boltz-2 affinity (0.62). By contrast, Boltz-2 confidence displays generally weaker agreement with other modalities (typically 0.51 to 0.60), indicating a more complementary signal. DrugBAN and MolTrans show moderate mutual concordance (0.57) and moderate agreement with the remaining modalities (often ≈0.51 to 0.61), with the lowest concordance observed between DrugBAN and Uni-Mol similarity (0.49). Overall, the concordance structure supports multimodal fusion: correlated predictors provide stability, while less concordant modalities (notably Boltz-2 confidence and parts of the interaction-based predictors) contribute additional ranking information.

### Consensus score performance across screening depths

Table 2 summarizes EFs and recovered held-out actives (hits) across standard virtual screening depths (5 repeated random splits; mean ± SD). The CWRA consensus score achieves the strongest very early enrichment and remains competitive as the screening budget increases. At a 1% screening depth, CWRA attains EF@1% = 21.32 ± 6.41 with 16 ± 5 recovered actives, substantially improving over equal-weight fusion (EF@1% = 10.79 ± 2.81; 8 ± 2 hits) and over the best single modality at this depth (GraphDTA ${\mathrm{IC}}_{50}$: EF@1% = 16.32 ± 3.96; 12 ± 3 hits). This advantage persists at a 2.5% screening depth, where CWRA remains best (EF@2.5% = 14.13 ± 2.34; 27 ± 4 hits) compared to equal-weight fusion (7.80 ± 1.22; 15 ± 2 hits) and GraphDTA ${\mathrm{IC}}_{50}$ (12.23 ± 1.35; 23 ± 3 hits).

**Table 2.** Extended virtual screening performance of individual scoring methods and fusion baselines on the VDR ligand discovery task (mean ± SD). *N* = 14,902 compounds (503 actives); 5 repeated random splits of actives (train fraction = 0.85); split seed = 42; drug-likeness prefilter removed 1,294/16,196 compounds; top *k* cutoffs: @1% = 149, @2.5% = 372, @5% = 745, @10% = 1,490, and @20% = 2,980. Hits, number of held-out active compounds retrieved. Bold, best; underlined, second best.

Method	Enrichment factor (EF)					Hits				
	@1%	@2.5%	@5%	@10%	@20%	@1%	@2.5%	@5%	@10%	@20%
GraphDTA $K_{d}$	10.79 ± 2.11	4.43 ± 0.86	2.58 ± 0.39	1.79 ± 0.18	1.30 ± 0.11	8 ± 2	8 ± 2	10 ± 1	14 ± 1	20 ± 2
GraphDTA $K_{i}$	0.00 ± 0.00	0.00 ± 0.00	0.21 ± 0.26	0.26 ± 0.14	0.29 ± 0.12	0 ± 0	0 ± 0	1 ± 1	2 ± 1	4 ± 2
GraphDTA ${\mathrm{IC}}_{50}$	16.32 ± 3.96	12.23 ± 1.35	8.32 ± 0.76	4.61 ± 0.55	2.72 ± 0.24	12 ± 3	23 ± 3	32 ± 3	35 ± 4	41 ± 4
MLT-LE ${\mathrm{pK}}_{d}$	2.90 ± 1.93	1.90 ± 0.54	1.58 ± 0.44	1.74 ± 0.24	1.41 ± 0.14	2 ± 1	4 ± 1	6 ± 2	13 ± 2	21 ± 2
TankBind	0.26 ± 0.53	0.53 ± 0.58	0.32 ± 0.26	0.16 ± 0.13	0.22 ± 0.15	0 ± 0	1 ± 1	1 ± 1	1 ± 1	3 ± 2
DrugBAN	3.16 ± 1.05	2.00 ± 0.70	1.68 ± 0.49	1.34 ± 0.21	1.41 ± 0.17	2 ± 1	4 ± 1	6 ± 2	10 ± 2	21 ± 3
MolTrans	0.26 ± 0.53	0.42 ± 0.21	0.63 ± 0.27	0.53 ± 0.30	0.58 ± 0.21	0 ± 0	1 ± 0	2 ± 1	4 ± 2	9 ± 3
AutoDock Vina	3.16 ± 1.05	1.48 ± 0.61	1.74 ± 0.59	2.08 ± 0.23	1.88 ± 0.23	2 ± 1	3 ± 1	7 ± 2	16 ± 2	29 ± 3
Boltz-2 affinity	1.05 ± 0.98	4.43 ± 1.27	5.47 ± 0.99	5.42 ± 0.74	4.04 ± 0.29	1 ± 1	8 ± 2	21 ± 4	41 ± 6	61 ± 4
Boltz-2 confidence	12.90 ± 3.16	11.81 ± 1.51	**11.05 ± 0.78**	**9.24 ± 0.26**	**5.00 ± 0.00**	10 ± 2	22 ± 3	**42 ± 3**	**70 ± 2**	**76 ± 0**
Uni-Mol	2.90 ± 1.53	3.90 ± 1.32	3.00 ± 0.83	2.42 ± 0.40	1.82 ± 0.24	2 ± 1	7 ± 2	11 ± 3	18 ± 3	28 ± 4
Equal-weight	10.79 ± 2.81	7.80 ± 1.22	6.32 ± 1.30	5.18 ± 0.52	3.21 ± 0.15	8 ± 2	15 ± 2	24 ± 5	39 ± 4	49 ± 2
Random-weight fusion	8.61 ± 1.38	6.34 ± 1.08	5.07 ± 0.64	4.36 ± 0.32	2.85 ± 0.14	6 ± 1	12 ± 2	19 ± 2	33 ± 2	43 ± 2
CWRA	**21.32 ± 6.41**	**14.13 ± 2.34**	9.47 ± 0.60	7.13 ± 0.72	4.18 ± 0.49	**16 ± 5**	**27 ± 4**	36 ± 2	54 ± 5	64 ± 7

VDR, vitamin D receptor; CWRA, Calibrated Weighted Rank Aggregation

At moderate depths (5% to 20%), Boltz-2 confidence becomes the strongest individual signal (EF@5% = 11.05 ± 0.78; 42 ± 3 hits; EF@10% = 9.24 ± 0.26; 70 ± 2 hits; EF@20% = 5.00 ± 0.00; 76 ± 0 hits), while CWRA remains consistently second best at these depths (EF@5% = 9.47 ± 0.60; 36 ± 2 hits; EF@10% = 7.13 ± 0.72; 54 ± 5 hits; EF@20% = 4.18 ± 0.49; 64 ± 7 hits). Overall, CWRA provides a robust consensus ranking: it maximizes early enrichment (1% to 2.5%) while retaining strong performance when screening sets are expanded. Across 100 repeated holdout splits, these gains are statistically significant: one-sided Nadeau–Bengio corrected resampled *t* tests (Holm-adjusted across the 5 cutoffs and 2 comparators) confirm that CWRA significantly exceeds both equal-weight fusion and the strongest single modality (GraphDTA ${\mathrm{IC}}_{50}$) at every screening depth from 1% to 20%, with the smallest—although still significant—margin over GraphDTA ${\mathrm{IC}}_{50}$ at the most stringent EF@1% cutoff (ΔEF=2.93, $P_{\text{Holm}}=0.048$) and progressively larger margins at broader depths (Section S2.16 and Table S12).

Beyond EF/hits, additional split-averaged ranking metrics show the same qualitative pattern: CWRA yields the strongest early-retrieval metric (BEDROC) and the highest AUPRC among fusion baselines, whereas Boltz-2 confidence achieves the highest AUROC (Supplementary Results). This separation is consistent with the intuition that a confidence-driven structural signal can yield strong global discrimination, while CWRA is explicitly optimized for early prioritization.

These gains are consistent with the BEDROC-optimized fusion objective, which explicitly emphasizes early recognition while still producing stable performance at moderate depths (e.g., 5% to 10%), making the consensus score suitable both for selecting a small, high-confidence follow-up set and for broader candidate coverage. As an internal sanity check, calcitriol (endogenous VDR ligand) is ranked no. 10 in the prefiltered pool of $N^{\prime }=14,902$ compounds (top 0.07%), indicating that CWRA appropriately prioritizes a canonical high-affinity binder.

### Learned modality weights and complementarity

CWRA learns a concentrated (but nondegenerate) weighting scheme: most of the mass is assigned to a small subset of consistently informative modalities, while the remaining signals are kept near the imposed lower bound (Table 3). The largest weights are assigned to GraphDTA ${\mathrm{IC}}_{50}$ (0.242 ± 0.002), AutoDock Vina (0.206 ± 0.036), and Boltz-2 confidence (0.155 ± 0.044), followed by DrugBAN (0.101 ± 0.014) and TankBind (0.069 ± 0.041). Boltz-2 affinity (0.053 ± 0.025), MolTrans (0.042 ± 0.010), GraphDTA $K_{d}$ (0.037 ± 0.008), and Uni-Mol (0.034 ± 0.004) receive smaller but nonzero contributions, while GraphDTA $K_{i}$ and MLT-LE are effectively at the lower bound (≈0.030). Sensitivity analyses with looser and tighter bounds showed similar enrichment performance and high top 100 overlap with the default ranking, indicating that the results are not critically dependent on the exact numerical bounds (Table S5).

**Table 3.** Fusion weights learned by CWRA and average rank position of active compounds (5 repeated random splits, mean ± SD)

Method	Weight	Mean rank
GraphDTA $K_{d}$	0.037 ± 0.008	6,523 ± 433
GraphDTA $K_{i}$	0.030 ± 0.001	12,335 ± 374
GraphDTA ${\mathrm{IC}}_{50}$	0.242 ± 0.002	4,682 ± 623
MLT-LE ${\mathrm{pK}}_{d}$	0.030 ± 0.000	5,881 ± 380
TankBind	0.069 ± 0.041	9,632 ± 91
DrugBAN	0.101 ± 0.014	6,577 ± 236
MolTrans	0.042 ± 0.010	10,085 ± 638
AutoDock Vina	0.206 ± 0.036	4,425 ± 331
Boltz-2 affinity	0.053 ± 0.025	2,217 ± 379
Boltz-2 confidence	0.155 ± 0.044	718 ± 42
Uni-Mol	0.034 ± 0.004	4,801 ± 347
CWRA	-	1,573 ± 682

CWRA, Calibrated Weighted Rank Aggregation

A weight-perturbation analysis further supported ranking robustness: additive perturbation of the learned CWRA weights followed by projection back onto the capped simplex retained 97% ± 1% of the top 100 compounds for Δ=1%, 89% ± 4% for Δ=5%, and 82% ± 5% even for the larger perturbation level Δ=10%, with corresponding top 2,000 Spearman correlations of 0.998 ± 0.002, 0.971 ± 0.024, and 0.907 ± 0.082, respectively (Table S4).

Importantly, the learned weights are not a simple reflection of unimodal performance measured by a single global statistic (e.g., mean rank). For example, Boltz-2 confidence yields the best unimodal mean rank of actives (mean rank 718 ± 42), yet CWRA assigns substantial mass to GraphDTA ${\mathrm{IC}}_{50}$ and Vina, consistent with the idea that these modalities provide complementary reorderings that improve early prioritization when fused. Consistent with this objective, CWRA improves the average rank of held-out actives (mean rank 1,573 ± 682) relative to most individual modalities (Table 3) while achieving the strongest EF@1% and EF@2.5% in Table 2. Overall, CWRA combines partially redundant high-signal modalities for stability while retaining bounded contributions from additional predictors, yielding a consensus ranking optimized for early enrichment rather than any single unimodal criterion.

### Generalization to an additional protein target

To test whether the observed performance was restricted to the VDR dataset, we applied the same CWRA workflow to an additional target, the GABA_A_ receptor. This target was included as a computational cross-target benchmark because it represents a protein class distinct from the nuclear receptor family. The analysis was limited to ranking performance, learned modality weights, and cross-target source-model transfer; detailed GABA_A_-specific structural or medicinal chemistry interpretation was outside the scope of the study. Curated experimental GABA_A_ binders served as positives, and a target-specific *de novo* generated library was used as the candidate pool. CWRA weights were learned and evaluated under the same repeated held-out-active protocol, drug-likeness filtering, 11-modality scoring pipeline, and split-honest Uni-Mol centroid computation used for VDR.

On GABA_A_, target-specific CWRA again achieved the strongest very early enrichment among the evaluated fusion and individual-modality baselines (Table 4). EF@1% reached 25.41 ± 3.96, compared with 17.47 ± 6.17 for equal-weight fusion and 11.64 ± 2.12 for the best individual modality at this cutoff, Boltz-2 confidence. CWRA also achieved the strongest performance at 2.5% and 5% screening depths (EF@2.5%=22.12±3.46 and EF@5%=14.33±0.84). At broader screening depths, AutoDock Vina became the strongest method (Table S9), consistent with CWRA being explicitly optimized for very early enrichment rather than uniform dominance across all cutoffs. These results address the single-target concern by showing that the same target-adaptive CWRA procedure can improve early enrichment on a second, mechanistically distinct protein target. A paired statistical significance analysis (Section S2.16 and Table S12) confirms that these GABA_A_ gains are statistically robust: CWRA significantly outperforms equal-weight fusion at every screening depth and the best single modality (Boltz-2 confidence) at all depths except 10% (ΔEF=−0.40, $P_{\text{Holm}}=0.97$), consistent with CWRA being optimized for very early rather than uniform enrichment.

**Table 4.** Cross-target evaluation of target-specific CWRA and strict source-model transfer (EF, mean ± SD over 5 held-out-active splits). VDR and GABA_A_ were evaluated using the same 11-modality CWRA pipeline with target-specific generated libraries and split-honest Uni-Mol centroid computation. For each target, target-specific CWRA gives the strongest very early enrichment, whereas strict transfer of the source-target CWRA model is weaker than target-specific recalibration. Full per-target results, modality weights, and transfer experiments are reported in the Supplementary Materials. Boldface indicates highest enrichment per target.

Target	Method	EF@1%	EF@2.5%	EF@5%
VDR	Best modality (GraphDTA ${\mathrm{IC}}_{50}$)	16.32 ± 3.96	12.23 ± 1.35	8.32 ± 0.76
VDR	Equal-weight fusion	10.79 ± 2.81	7.80 ± 1.22	6.32 ± 1.30
VDR	GABA_A_ source model → VDR	9.74 ± 1.34	8.75 ± 0.63	6.26 ± 1.08
VDR	CWRA	**21.32 ± 6.41**	**14.13 ± 2.34**	**9.47 ± 0.60**
GABA_A_	Best modality (Boltz-2 confidence)	11.64 ± 2.12	10.32 ± 2.04	9.69 ± 0.98
GABA_A_	Equal-weight fusion	17.47 ± 6.17	12.64 ± 1.76	10.22 ± 1.78
GABA_A_	VDR source model → GABA_A_	9.53 ± 3.96	8.01 ± 1.58	6.74 ± 1.35
GABA_A_	CWRA	**25.41 ± 3.96**	**22.12 ± 3.46**	**14.33 ± 0.84**

CWRA, Calibrated Weighted Rank Aggregation; EF, enrichment factor; VDR, vitamin D receptor; GABA_A_, γ-aminobutyric acid type A

The learned weights were strongly target specific yet interpretable (Table S10). For VDR, the highest weights were assigned to GraphDTA ${\mathrm{IC}}_{50}$, AutoDock Vina, Boltz-2 confidence, and DrugBAN, whereas GABA_A_ shifted weight toward Uni-Mol, DrugBAN, AutoDock Vina, and GraphDTA $K_{i}$. This indicates that CWRA does not learn a universal modality hierarchy but instead calibrates the relative usefulness of each modality for the target under study. To test whether the learned source models themselves transfer without target-specific recalibration, we performed a strict cross-target source-model transfer experiment in both directions. In this setting, source-target weights, source normalization ranges, and source Uni-Mol centroids were applied unchanged to the destination target, and destination labels were used only for held-out enrichment evaluation. Strict transfer retained above-random enrichment but was substantially weaker than target-specific CWRA: GABA_A_ source models transferred to VDR achieved EF@1%=9.74±1.34 versus 21.32 ± 6.41 for target-specific VDR CWRA, while VDR source models transferred to GABA_A_ achieved EF@1%=9.53±3.96 versus 25.41 ± 3.96 for target-specific GABA_A_ CWRA (Table S11). Thus, CWRA should be interpreted as a transferable target-adaptive calibration framework, not as a universal fixed-weight model. This directly addresses the risk of target-specific overfitting by showing that recalibration on held-out-active splits is essential and that the learned weights encode target-dependent modality informativeness rather than a globally transferable ranking rule.

### Structural motif enrichment in CWRA-ranked candidates

**Fig. 4.**
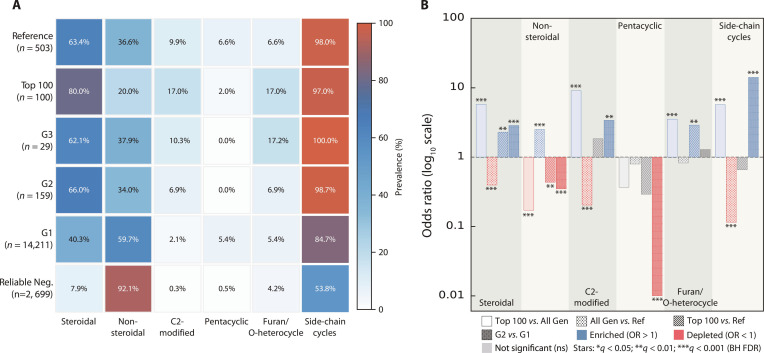
Vitamin D receptor (VDR)-relevant structural motifs are progressively enriched from single-generator candidates to the Calibrated Weighted Rank Aggregation (CWRA)-ranked short list. (A) Prevalence (%) of 6 predefined structural features across compound groups: known VDR reference actives (Reference, n=503), the CWRA-ranked unlabeled short list (Top 100, n=100), generated candidates stratified by cross-generator overlap (G1 to G3, corresponding to molecules produced by exactly 1, 2, or ≥3 generators), the union of all generated candidates (All Gen), and reliable negatives (RNs; n=2,699). Color encodes prevalence from white (0%) to dark blue or red (100%), with blue shading indicating secosteroidal character and red shading indicating nonsteroidal character. (B) Feature-wise enrichment and depletion across 4 pairwise comparisons (Top 100 *vs.* All Gen; All Gen *vs.* Ref; Top 100 *vs.* Ref; G2 *vs.* G1), reported as odds ratios (OR; log_10_ scale; OR > 1, enriched in blue; OR < 1, depleted in red). For each feature–comparison pair, a 2 × 2 contingency table (feature present/absent × group) was constructed and a 2-sided Fisher’s exact test applied; *P* values are corrected for multiple testing using the Benjamini–Hochberg false discovery rate (FDR) procedure, and significance is annotated as * (q<0.05), ** (q<0.01), and *** (q<0.001); nonsignificant comparisons are shown in gray.

Figure 4 summarizes the distribution of 6 structural features across the reference actives, generated pools, and the CWRA-ranked short list. Notably, the top 100 short list is enriched for secosteroidal scaffolds and C2α-modified secosteroids, structural motifs with extensively documented roles in VDR binding affinity and agonist potency [16,17] without any explicit structural constraints imposed during either generation or scoring.

#### Secosteroidal and nonsteroidal scaffolds

Secosteroidal prevalence increases from 40.3% in single-generator molecules (G1) to 66.0% in 2-generator consensus molecules (G2) and 80.0% in the CWRA top 100, compared to 63.4% in the reference active set (Fig. 4A). Each pairwise comparison is significant (All Gen *vs.* Ref: q<0.001; G2 *vs.* G1: q<0.001; Top 100 *vs.* All Gen: q<0.001; Top 100 *vs.* Ref: q<0.01; Fig. 4B). Nonsteroidal prevalence is correspondingly depleted at every level. RNs show the opposite profile (92.1% nonsteroidal; 7.9% secosteroidal; Fig. 4A). Representative highest-scoring nonsteroidal candidates are provided in Fig. S1 together with their CWRA scores, conformal *P* values, generator-overlap class, and selected physicochemical descriptors.

#### C2α-modified secosteroids

C2α-modified secosteroid prevalence rises from 2.1% in G1 to 6.9% in G2, 10.3% in G3, and 17.0% in the CWRA top 100, compared to 9.9% in the reference (Fig. 4A). The G2 *vs.* G1 (q<0.01) and Top 100 *vs.* All Gen (q<0.001) comparisons are significant (Fig. 4B), indicating that both cross-generator consensus and CWRA ranking enrich for this motif relative to the broader generated pool.

#### Pentacyclic scaffolds

Pentacyclic motifs are present in 6.6% of reference actives but are strongly depleted in the top 100 (2.0%; Top 100 *vs.* Ref: q<0.001; Fig. 4A and B).

#### Furan/O-heterocycle motifs

Furan and O-heterocyclic elements are enriched in the top 100 (17.0%) relative to both the reference (6.6%; q<0.001) and the full generated pool (q<0.001) and are similarly elevated among G3 consensus molecules (17.2%; G2 *vs.* G1: q<0.001; Fig. 4A and B).

### Post hoc interaction analysis of Boltz-2 affinity predictions with PLIP

**Fig. 5.**
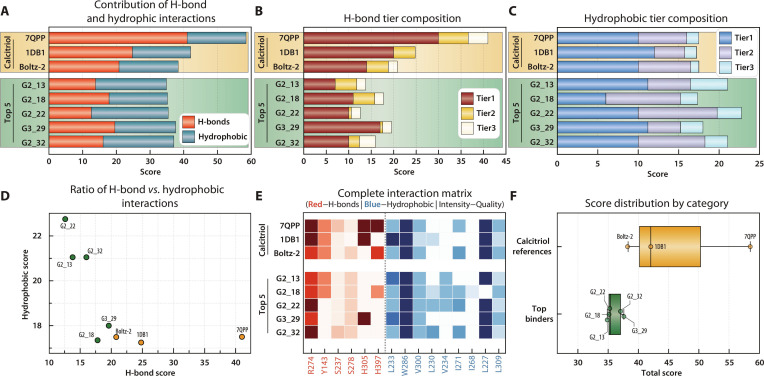
Post hoc interaction analysis of calcitriol references and 5 top-ranked generated compounds based on Boltz-2-predicted structures. (A) Relative contribution of hydrogen-bond and hydrophobic interactions to the total Protein–Ligand Interaction Profiler (PLIP) interaction score for 3 calcitriol reference structures (7QPP, 1DB1, and Boltz-2 predicted) and 5 top-ranked generated compounds (G2_13, G2_18, G2_22, G3_29, and G2_32). (B) Hydrogen-bond tier composition showing distribution of Tier1, Tier2, and Tier3 interactions across all analyzed structures. (C) Hydrophobic tier composition across interaction quality tiers. (D) Ratio of hydrogen-bond versus hydrophobic interaction scores; orange points indicate calcitriol references, and green points indicate top-ranked generated compounds. (E) Residue-level interaction matrix showing hydrogen-bond (red) and hydrophobic (blue) contacts with 15 key ligand-binding pocket (LBP) residues; color intensity reflects interaction quality. Residues left of the dashed line are hydrogen-bond donors/acceptors; residues right of the dashed line are hydrophobic contacts. (F) Total PLIP interaction score distribution across calcitriol references and top-ranked generated compounds.

**Fig. 6.**
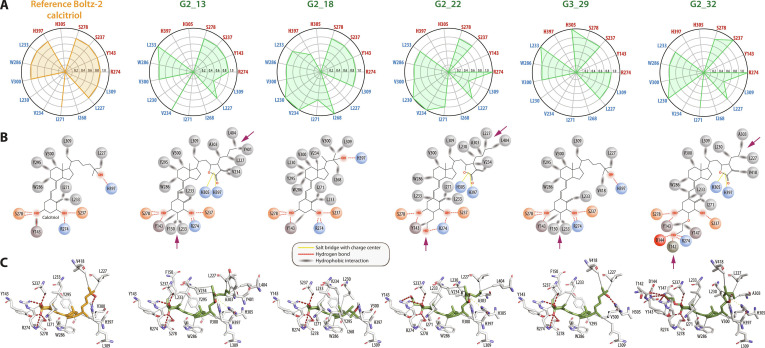
Interaction fingerprints and structural representations for the Boltz-2 calcitriol reference structure and 5 top-ranked generated compounds. (A) Radial interaction fingerprints summarizing hydrogen-bond (red residue labels) and hydrophobic (blue residue labels) interactions with curated vitamin D receptor (VDR) ligand-binding pocket residues. Radial distance represents the tier-weighted per-residue Protein–Ligand Interaction Profiler (PLIP) interaction score (Tier1 × 1.0 + Tier2 × 0.6 + Tier3 × 0.3), normalized to [0,1] by dividing by the maximum per-residue score across all 6 analyzed structures (calcitriol reference structure and 5 generated compounds). Normalization was performed separately for hydrogen-bond and hydrophobic interaction residue sets. Columns from left to right: Boltz-2 calcitriol reference, G2_13, G2_18, G2_22, G3_29, G2_32. (B) Two-dimensional interaction schematics for the same 6 structures. Red dashed lines indicate hydrogen bonds, yellow lines indicate salt bridges with charge center, and gray shading indicates hydrophobic contacts. Violet arrows indicate interactions beyond the canonical calcitriol contact set. (C) Three-dimensional PLIP interaction diagrams for Boltz-2-predicted structures. Red dashed lines indicate hydrogen bonds, yellow lines indicate salt bridges with charge center, and gray lines indicate hydrophobic contacts.

To characterize the interaction profiles of CWRA-prioritized compounds, we performed post hoc protein–ligand interaction analysis using PLIP [34] on Boltz-2-predicted structures. Analysis included 3 calcitriol reference structures (low-resolution x-ray structure 1DB1, high-resolution x-ray structure 7QPP, and a Boltz-2-predicted calcitriol structure) and the 5 top-ranked generated compounds according to CWRA consensus score (G2_13, G2_18, G2_22, G3_29, and G2_32; Figs. 5 and 6).

The 3 calcitriol reference structures showed consistent interaction profiles across hydrogen-bond and hydrophobic contacts with key LBP residues (Fig. 5A to E). The 7QPP structure displayed the highest total PLIP score with balanced hydrogen-bond and hydrophobic contributions and predominance of Tier1 hydrogen bonds at canonical residues R274, Y143, S237, S278, H305, and H397 (Fig. 5B and E). Structure 1DB1 showed a similar pattern with slightly reduced hydrogen-bond scores. The Boltz-2-predicted calcitriol structure produced intermediate hydrogen-bond scores with mixed tier composition and substantial hydrophobic character (Fig. 5A to D).

The 5 structurally diverse top-ranked generated compounds represent 3 distinct structural classes relative to the canonical calcitriol scaffold, all retaining the 1α,3β-diol A-ring and the 19-methylene group. G3_29 is a C2α-methyl-calcitriol analog, directly corresponding to the methyl-substituted class characterized by Hourai *et al.* [17], retaining the canonical 25-hydroxylated side chain. G2_18 is a 22,23,24-trinor analog with a 3-carbon deletion in the aliphatic side chain, a conserved terminal hydroxyl group, and no C2α-modification. G2_13, G2_22, and G2_32 each incorporate a 25-deoxy 23,26-γ-lactone in the side chain, belonging to the TEI-9647-related lactone series previously characterized as VDR modulators [36]. G2_13 bears a C2α-methyl A-ring substituent and a 4-butyl group at C24 of the lactone ring, G2_22 bears a C2α-(3-hydroxypropyl) A-ring substituent and a 4-butyl group at C24, and G2_32 bears a C2α-(2-hydroxyethoxy) A-ring substituent and a 4-propyl group at C24. The C2α-functionalization patterns of G2_13, G2_22, and G2_32 closely parallel the C2α-methyl, 3-hydroxypropyl, and 3-hydroxypropoxy series reported by Saito *et al.* [36], with G2_22 and G2_32 representing the 2-carbon homologs of the latter 2 classes (Fig. 6B and C).

The total PLIP scores of all 5 top-ranked compounds fall within or near the range observed for the calcitriol references (Fig. 5F), with hydrophobic interactions dominating the total score across all structures (Fig. 5A and C). Hydrogen-bond networks show predominantly Tier1 character for compounds retaining canonical A-ring hydroxyl groups (Fig. 5B). A notable interaction feature distinguishes the 3 lactone-containing compounds (G2_13, G2_22, and G2_32) from the remaining structures, because PLIP reports salt bridges with charge center rather than classical hydrogen bonds at H305 and H397 (Fig. 6B, yellow lines), with the lactone carbonyl oxygens simultaneously coordinating both histidine residues. This contrasts with calcitriol, G2_18, and G3_29, where classical hydrogen bonds are formed at these residues via the 25-hydroxyl group. The lactone carbonyl thus replaces the 25-OH hydrogen-bond donor/acceptor function through a distinct electrostatic interaction mode.

Residue-level interaction analysis (Fig. 5E) shows that all 5 top-ranked compounds engage the canonical A-ring hydrogen-bond residues R274, Y143, S237, and S278, consistent with the presence of 1α,3β-diol functional groups across all 5 structures. Hydrophobic contacts are distributed across multiple LBP residues including L233, W286, V300, L230, V234, I271, I268, L227, and L309, partially reproducing the calcitriol hydrophobic contact pattern (Fig. 5E). Radial interaction fingerprints (Fig. 6A) are consistent with broad LBP coverage by all 5 top-ranked compounds, with fingerprint areas approaching or matching the Boltz-2 calcitriol reference across both hydrogen-bond and hydrophobic residues, although these geometries remain computational predictions. Individual compounds show distinct fingerprint shapes reflecting their structural differences such as G3_29 most closely mirroring the calcitriol reference pattern given its C2α-methyl modification on an otherwise canonical scaffold, G2_18 showing a slightly reduced fingerprint in the side-chain hydrophobic sector consistent with its shortened aliphatic chain, and G2_13, G2_22, and G2_32 showing expanded contacts at residues outside the canonical calcitriol set (violet arrows, Fig. 6B and C) attributable to their C2α A-ring substituents and the 25-deoxy C24-alkylated 23,26-γ-lactone in the side chain.

Two-dimensional interaction schematics (Fig. 6B) and 3D PLIP diagrams (Fig. 6C) are consistent with deep burial of all 5 compounds within the LBP, with ligand poses suggesting occupation of the canonical calcitriol binding volume; experimental structural validation would be required to confirm these binding geometries.

## Discussion

Traditional consensus approaches often rely on equal weighting or ad hoc combinations that do not adapt to specific targets [6,7]. In contrast, CWRA learns modality weights by measuring how well each modality helps recover known VDR binders near the top of the ranked list and then forms a consensus score as a convex combination of per-modality normalized scores. Normalization mitigates scale differences across heterogeneous physics-based and ML predictors ($N^{\prime }=14,902$ compounds, *A* = 503 known actives), while the rank-based BEDROC objective ensures that weight learning targets early recognition. As a sensitivity analysis, we repeated VDR cross-validation using fold-isolated normalization, in which held-out test actives were excluded from estimation of normalization parameters; enrichment estimates were essentially unchanged (Section S2.9).

Calibration led to consistent enrichment gains across the screening depths typically used to select compounds for experimental testing (Table 2), consistent with recent virtual screening approaches [37].

This direct approach offers several advantages. First, the objective directly maximizes early recognition on the same candidate pool used for scoring by optimizing the BEDROC metric (with α=100), which assigns exponentially higher importance to the very top of the ranked list and therefore aligns closely with practical screening goals. This avoids the need to tune ad hoc intermediate calibration parameters or to optimize a collection of separate EF cutoffs. Second, DE effectively handles the nonconvex, combinatorial nature of rank-based objectives, reducing sensitivity to local optima. Third, the bound constraints integrate naturally into the optimization framework, ensuring that every candidate solution satisfies the explicit per-modality weight-bound constraints.

CWRA is conceptually related to supervised score-fusion and learning-to-rank approaches, since it learns a weighted combination of modality scores. Its contribution is not a new neural ranking architecture, but a virtual-screening-specific formulation: BEDROC-based optimization directly targets very early enrichment, the objective can be trained using confirmed actives without assuming that unlabeled compounds are true inactives, and bounded simplex constraints yield interpretable modality weights while preventing single-modality collapse. Direct comparison to gradient-boosted or neural ranking models would require curated negative or graded relevance labels; in the present PU screening setting, such labels are unavailable, and constructing them heuristically would introduce the same reliable-negative uncertainty discussed in the limitations below. We therefore view systematic benchmarking against modern learning-to-rank methods on datasets with confirmed inactive compounds as an important direction for future work.

The simplicity of the CWRA formulation, i.e., BEDROC-driven early-recognition optimization under explicit bound constraints, facilitates transfer to new targets and modality combinations. Users need only specify the admissible weight ranges (and, if desired, the BEDROC sharpness parameter *α*); all remaining details are handled automatically by the optimization procedure.

We tested this transferability directly. Applying CWRA to the mechanistically distinct GABA_A_ receptor provided an additional test of whether the framework could be recalibrated beyond the primary VDR application. On both VDR and GABA_A_, target-specific calibration produced the strongest very early enrichment (Section S2.15), supporting the methodological portability of CWRA beyond VDR. These experiments also address the concern that learning weights from known binders on the same target could lead to target-specific overfitting. First, enrichment was evaluated only on held-out actives, and the one modality that directly compares candidates with the active set, Uni-Mol similarity, was computed split-honestly, with the reference centroid recalculated using only the training-split actives (see the “CWRA meta-score for multimodal ranking” section). Second, the dominant modality weights were stable across folds but differed between VDR and GABA_A_, indicating target-dependent modality informativeness rather than a universal fixed weighting pattern. Third, strict cross-target source-model transfer was evaluated in both directions: source-target weights, normalization ranges, and Uni-Mol centroids were applied unchanged to the destination target, with destination labels used only for held-out enrichment evaluation. These transferred source models retained above-random early enrichment but were substantially weaker than target-specific recalibration, indicating that CWRA should be interpreted as a transferable target-adaptive calibration framework rather than a universal fixed-weight model. Because only one additional target was examined and no detailed GABA_A_-specific chemical interpretation was undertaken, these findings should be regarded as evidence of methodological portability rather than comprehensive validation across protein families. As a complementary within-target analysis, we report Bemis–Murcko scaffold-level novelty of the generated library (Table 1), in which CWRA preferentially recovered reference-associated VDR scaffold classes from a library that was 98.8% scaffold-novel relative to the reference actives.

The 11 scoring modalities provide complementary information. Concordance analysis (Fig. 3) and pairwise modality comparisons (Fig. 2) show moderate agreement between most pairs. The strongest overlap occurs within GraphDTA endpoints (e.g., $K_{d}$ *vs.* $K_{i}$), while Boltz-2 confidence is more independent from affinity-based predictors.

As can be seen in the Venn diagram (Fig. 1), the 3 fine-tuned generators explore distinct regions of VDR chemical space with only a small shared intersection. Using multiple generator architectures (e.g., recurrent neural network and transformer based) increases the chance of producing candidates that score well across different evaluation modalities.

The Bemis–Murcko scaffold analysis suggests that the generated library is not merely a scaffold-level reproduction of the 503 reference binders, since 98.8% of generated scaffolds were absent from the reference scaffold set (see Table 1). However, the CWRA top 100 candidates were strongly enriched for reference-like scaffolds, reflecting the BEDROC-based objective that prioritizes early recovery of known VDR-active chemotypes. The top 100 scaffold profile more closely resembles the G3 consensus class than the G1 single-generator class, indicating that cross-generator agreement already biases the candidate pool toward reference-like chemistry prior to CWRA ranking. The BEDROC-calibrated prioritization step then further sharpens this enrichment toward VDR-compatible scaffold classes. We therefore interpret the *de novo* workflow as combining scaffold-diverse generation with conservative, VDR-calibrated prioritization. The 2 reference-novel scaffolds in the top 100 should be viewed as exploratory hypotheses for follow-up validation rather than experimentally confirmed novel VDR chemotypes.

The post hoc structural and interaction analysis indicates 2 levels of convergence that together support CWRA’s ranking behavior and the ML-guided discovery pipeline, spanning population-level enrichment of privileged VDR scaffolds across the generative pools and individual-compound recovery of historically established medicinal chemistry at the top of the ranked list.

The structural feature analysis (Fig. 4) reveals that C2α-modified secosteroids and 23,26-lactone derivatives, 2 privileged VDR scaffold classes, are rediscovered by the generative pipeline disproportionately to their representation in the training data. C2α-modified compounds account for only 9.9% of the 503 reference VDR binders used for fine-tuning, yet their prevalence rises to 17.0% in the CWRA top 100 and is significantly enriched in the multigenerator consensus pools (G2: 6.9%; G3: 10.3%; G2 *vs.* G1: q<0.001). This disproportionate enrichment is unlikely to arise from simple frequency matching during fine-tuning. A more plausible explanation is that C2α-modified and lactone-containing compounds are systematically overrepresented among the highest-affinity binders in the training data, leading the generative models to implicitly associate these structural features with high-activity examples and amplify minority scaffolds that carry strong binding signal. This effect is further supported by cross-generator convergence: architecturally diverse models independently generate the same minority scaffolds, providing evidence that these features are genuinely encoded as high-probability VDR-compatible structures rather than arising from stochastic sampling of frequent SMILES patterns. CWRA provides an additional layer of amplification by fusing 11 heterogeneous modalities that each independently score these compounds favorably, further concentrating C2α-modified and lactone derivatives at the top of the ranked list. The progressive enrichment from G1 (2.1% C2α-modified) through G2 (6.9%), G3 (10.3%), and top 100 (17.0%) thus reflects 3 successive filters. Generative fine-tuning, cross-generator consensus, and multimodal normalized-score fusion each independently amplify the same privileged structural signal.

**Fig. 7.**
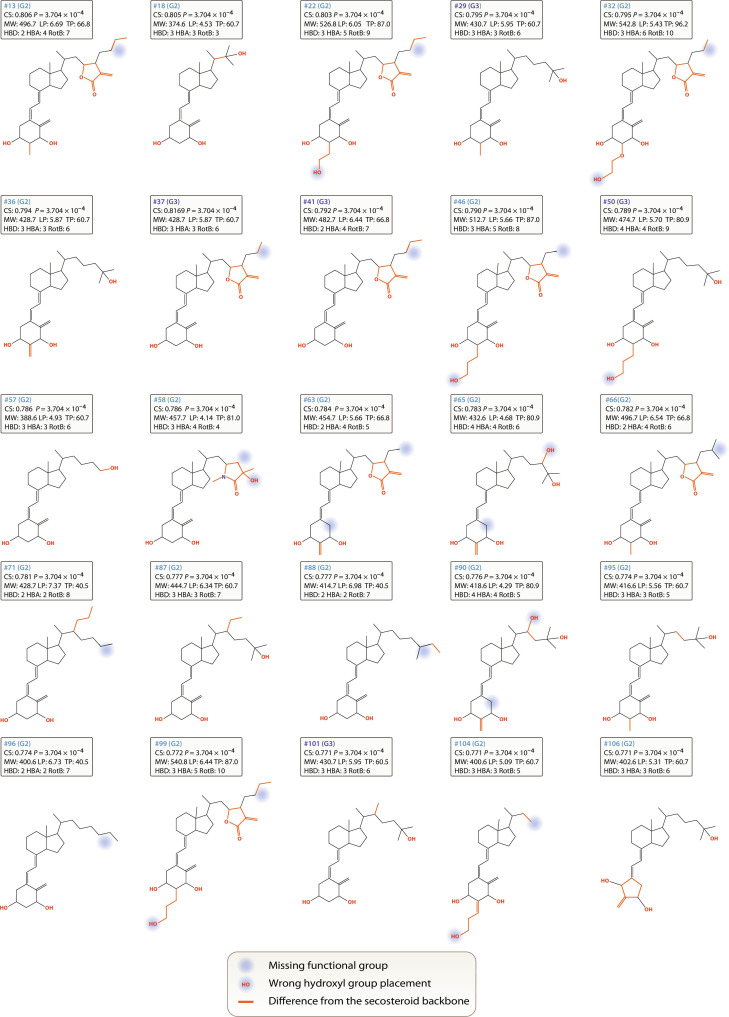
Top 25 *in silico* generated candidates ranked by Calibrated Weighted Rank Aggregation (CWRA_. Each cell shows the compound ID, generation source (G2 and G3), CWRA score with *P* value, molecular properties (MW, molecular weight; LP, LogP; TP, topological polar surface area; HBD, number of hydrogen-bond donors; HBA, number of hydrogen-bond acceptors; RotB, number of rotatable bonds). Structural differences from the canonical secosteroid backbone and functional group placing in calcitriol are highlighted in orange, blue shading indicates missing functional groups, and red “HO” labels in blue shading denote misplaced hydroxyl groups. Compound IDs denote each candidate’s rank within the full CWRA-ranked pool, which includes the 503 known vitamin D receptor (VDR) actives; since the top of the ranked list is dominated by known actives, the 25 top-ranked *de novo* candidates carry nonconsecutive IDs (here no. 13 to no. 106).

The 5 top-ranked compounds confirm and extend this population-level rediscovery of privileged scaffolds with precise chemical identity (Fig. 7). Four of the 5 top-ranked compounds (G3_29, G2_13, G2_22, and G2_32) bear C2α-substituents on the secosteroid A-ring. G2_18 is the exception, being a 22,23,24-trinor analog with a 3-carbon deletion in the aliphatic side chain, retaining the terminal hydroxyl group and the canonical A-ring without C2α-modification, representing a nor-series analog of established interest in VDR pharmacology [38]. G2_13, G2_22, and G2_32 belong to the TEI-9647-related 25-deoxy C24-alkylated 23,26-γ-lactone series [36], among the most potent VDR antagonists described in the medicinal chemistry literature, with the most active analog reaching ${\mathrm{IC}}_{50}=7.4\;\mathrm{pM}$ to inhibit differentiation of HL-60 cells induced by calcitriol [36]. G2_13 bears a C2α-methyl and G2_22 a C2α-(3-hydroxypropyl) A-ring substituent, both with a C24 4-butyl lactone, and G2_32 bears a C2α-(2-hydroxyethoxy) A-ring substituent with a C24 4-propyl lactone. The C2α-functionalization patterns of G2_13, G2_22, and G2_32 closely parallel the C2α-methyl, 3-hydroxypropyl, and 3-hydroxypropoxy series reported by Saito *et al.* [36], with G2_22 and G2_32 representing 2-carbon homologs of the latter 2 classes. That generative models trained only on known VDR binders, without explicit structural rules, independently converge on both C2α-modifications and the TEI-9647 lactone scaffold represents substantive evidence that the ML-guided discovery pipeline recovers established VDR pharmacophore classes.

PLIP analysis of Boltz-2-predicted structures suggests that all 5 top-ranked compounds adopt interaction profiles within or close to the range of calcitriol reference structures (Fig. 5F), with hydrophobic contacts distributed broadly across the LBP and hydrogen-bond networks at canonical A-ring anchoring residues R274, Y143, S237, and S278 (Fig. 5E), consistent with noncanonical yet structurally rationalized interaction profiles. The 3 lactone-containing compounds (G2_13, G2_22, and G2_32) engage H305 and H397 through salt bridges with charge center via the lactone carbonyl oxygens rather than through classical hydrogen bonds as calcitriol does via its 25-hydroxyl group (Fig. 6B, yellow lines), consistent with the known capacity of carbonyl groups to engage histidine residues through electrostatic geometry [35].

The apparent reduction in PLIP scores for the top-ranked generated compounds relative to the high-resolution calcitriol crystal structure (7QPP) reflects 2 distinct phenomena. First, Boltz-2 underestimates hydrogen-bond formation relative to crystallographic accuracy [10], systematically reducing scores for all predicted structures including the Boltz-2 calcitriol reference itself. Second, the generated compounds have C2α-substituent and C24-alkylated lactone ring functional groups that engage residues beyond the canonical calcitriol contact set, including V418, F150, Y401, A303, and D144 depending on the compound (Fig. 6B and C, violet arrows). Whether these alternative contacts contribute net favorably to binding affinity, and whether the lactone salt-bridge mode confers an agonist or an antagonist character, remains to be established experimentally. The rediscovery of both the C2α-modification series [17] and the highly potent TEI-9647 lactone series [36] in the top-ranked output demonstrates that CWRA can identify scaffolds with documented VDR pharmacophore patterns, providing a substantive foundation for experimental follow-up; whether specific analogs retain high-affinity binding requires experimental confirmation.

### Limitations of the current study

Several limitations of the present study merit consideration. First, the generative models were fine-tuned on 503 reference VDR binders, a training set that, while curated, does not represent the full chemical space of known VDR ligands and may bias generative output toward scaffolds already present or structurally similar to those in the reference data. The CWRA weight calibration likewise relies on reference-set performance; although target-specific recalibration improved very early enrichment on an additional, mechanistically distinct target, GABA_A_ (see the “Generalization to an additional protein target” section), the learned weights remained target specific and strict cross-target source-model transfer was substantially weaker than target-specific recalibration. Thus, CWRA should be interpreted as a transferable target-adaptive framework rather than a universal fixed-weight model, and calibration may not generalize with equal fidelity to scaffolds or targets that deviate substantially from the training distribution.

Second, RNs were used exclusively for empirical *P*-value calibration and diagnostic checks and were not used during CWRA weight optimization. Because these compounds are selected heuristically from unlabeled molecules, they should be interpreted as an approximate lower-tail null rather than experimentally confirmed nonbinders. Consequently, conformal *P* values are used here as empirical prioritization measures, and validation against benchmark datasets with confirmed inactive compounds remains an important future direction.

Third, generated molecules were represented, standardized, and selected as nonstereospecific SMILES and therefore at the level of constitutional molecular graphs rather than fully stereochemistry-resolved ligand structures. This is particularly relevant for VDR, where the spatial orientation of hydroxyl groups and the aliphatic side chain strongly influences ligand recognition. Accordingly, the top-ranked generated candidates should not be interpreted as immediately synthesis-ready stereochemical proposals. Before experimental prioritization, they require stereoisomer enumeration, stereochemistry-aware redocking or complex prediction, and expert chemical inspection.

Fourth, all protein–ligand interaction profiling was performed on Boltz-2-predicted structures rather than experimentally determined coordinates. Boltz-2 is known to underestimate hydrogen-bond formation relative to crystallographic accuracy [10], which systematically affects PLIP scores across all predicted structures and limits the quantitative interpretation of interaction tier assignments. PLIP interaction scores themselves are geometry-based descriptors rather than binding free energies and do not account for entropic contributions, desolvation penalties, or ligand conformational flexibility. Consequently, score comparisons between compounds should be interpreted as qualitative support for plausible binding modes rather than quantitative rankings of binding competence.

Finally, the 3 lactone-containing compounds (G2_13, G2_22, and G2_32) belong to a scaffold class documented primarily as VDR antagonists [36]. Whether these generated analogs act as agonists or antagonists, whether the predicted salt-bridge interaction mode at H305 and H397 is reproduced experimentally, and whether the biologically preferred stereoisomer has been uniquely identified all require biochemical and structural validation.

## Conclusion

In this work, we introduced CWRA, a practical rank-optimized score-fusion framework for multimodal virtual screening that learns nonnegative modality weights by optimizing early recognition under explicit per-modality weight-bound constraints. By direction-correcting heterogeneous modality outputs, normalizing them to a common scale, and optimizing a bounded-simplex weight vector (to prevent degenerate single-modality solutions), CWRA provides a simple and transferable alternative to ad hoc consensus rules.

Applied to VDR ligand discovery, CWRA fused 11 diverse modalities spanning docking, structure-based predictors, deep-learning affinity models, and ligand similarity. On a unified screening table of N=16,196 compounds containing P=503 known actives, we evaluated performance with 5 repeated random splits on a drug-likeness prefiltered pool of $N^{\prime }=14,902$ candidates. CWRA achieved strong very early enrichment on held-out actives (EF@1%=21.32±6.41, corresponding to 16 ± 5 recovered actives within the top 1% of the ranked list) and substantially outperformed both equal-weight fusion (EF@1%=10.79±2.81; 8 ± 2 hits) and the strongest single modality at this depth (GraphDTA ${\mathrm{IC}}_{50}$; EF@1%=16.32±3.96; 12 ± 3 hits). As an internal sanity check, calcitriol (endogenous VDR ligand) is ranked no. 10 in the prefiltered pool (top 0.07%), indicating that the consensus score appropriately prioritizes a canonical high-affinity binder.

Beyond aggregate performance, cross-modality analyses supported the central motivation for multimodal fusion: many modality pairs show only moderate agreement, with the strongest overlap among related GraphDTA endpoints, while structural confidence from Boltz-2 provides a complementary signal to affinity-oriented predictors. Finally, structural feature analyses of the CWRA-ranked short list indicate enrichment of VDR-relevant chemotypes including secosteroidal scaffolds and C2α-modified secosteroids that are extensively validated in the VDR medicinal chemistry literature. This convergence, obtained without explicitly encoding structure–activity rules, illustrates how calibrated multimodal consensus can amplify consistent signals across heterogeneous predictors and surface chemically meaningful candidates for experimental follow-up.

These results suggest that CWRA is a robust and interpretable approach for combining modern docking-, structure-, and ML-based modalities when prioritizing generative libraries. Future work will focus on prospective experimental validation of prioritized candidates, extending CWRA to additional targets and modality sets, and strengthening generalization assessment (e.g., via external benchmarks and stricter holdout calibration) to further reduce the risk of target-specific or dataset-specific bias.

## Data Availability

**Code:** The CWRA implementation and PU conformal selection pipeline used in this study are available at https://github.com/Salimzhanov/cwra-vdr (release v1.1.0). The repository includes reproduction instructions (README), environment specifications, and scripts to regenerate the main tables and figures. **Data and materials:** Curated datasets and derived outputs used in this study (including the standardized reference binder set, generated candidate libraries, modality outputs, and final ranked lists) are archived on Zenodo: zenodo.org/records/18022524. Large artifacts (e.g., model checkpoints and intermediate outputs) are provided alongside the archive where applicable. **License:** The code is released under the license specified in the GitHub repository. Data reuse is governed by the terms specified in the Zenodo record. **Reproducibility note:** All experiments were run with fixed random seed (42). RDKit was used for SMILES parsing, fingerprint similarity, and physicochemical descriptor computation [24].

## Supplementary Materials

Supplementary 1 Supplementary Methods Supplementary Results Fig. S1 Tables S1 to S12
