# Supplementary material for: Calibrated Weighted Rank Aggregation for Virtual Screening Independently Rediscovers Privileged Vitamin D Receptor Ligand Scaffolds
Source: Comput Struct Biotechnol J. 2026 Jul 13;35(1):0155. doi: 10.34133/csbj.0155 (PMC13358164; doi:10.34133/csbj.0155)
Supplement: Supplementary 1 — Supplementary Methods Supplementary Results Fig. S1 Tables S1 to S12 [file csbj.0155.f1.pdf]

Supplementary Information for
Calibrated Weighted Rank Aggregation for Virtual Screening Independently Rediscovers Privileged Vitamin D Receptor Ligand Scaffolds

Abylay Salimzhanov[1], Askar Boranbayev[1], Ferdinand Molnár*[2], and Siamac Fazli*[1]

[1]Department of Computer Science, School of Digital Sciences and Engineering, Nazarbayev University, Kabanbay Batyr Ave 53, Astana, Kazakhstan
[2]Department of Biology, School of Sciences and Humanities, Nazarbayev University, Kabanbay Batyr Ave 53, Astana, Kazakhstan

1 Methods

1.1 Generative models

1.1.1 REINVENT

REINVENT is a molecular generator that produces novel, drug-like compounds by learning over SMILES strings and optimizing molecular generation through deep reinforcement learning [1]. The model operates directly in the discrete space of SMILES sequences and updates a recurrent neural network to maximize a user-defined reward. The framework consists of two recurrent networks: a *Prior*, pretrained on a large corpus of bioactive molecules, and an *Agent*, initialized from the Prior and subsequently fine-tuned to optimize a chosen scoring function.

The underlying architecture is a recurrent neural network. SMILES strings are tokenized into individual symbols, mapped to integer indices, and passed through an embedding layer of dimension 128, which projects each token into a continuous vector representation. The embedded sequences are then processed by a stack of three gated recurrent unit (GRU) layers, each with 512 hidden units. A final linear layer followed by a softmax activation produces a categorical distribution over the next token at each time step, thereby defining an autoregressive generative model over SMILES strings. The Prior is trained by maximum-likelihood estimation on the ChEMBL dataset [2].

*Corresponding authors: ferdinand.molnar@nu.edu.kz, siamac.fazli@nu.edu.kz

Sequences are sampled from the Agent, evaluated with the scoring function, and used to update the Agent so as to increase the expected reward. REINVENT uses an augmented log-likelihood framework: for each generated sequence, the Prior and Agent log-likelihoods are computed, and the scalar reward is incorporated into the loss through the augmented objective

$$\mathcal{L} = \mathbb{E}\left[(\log p_{\text{Agent}}(\mathbf{s}) - \log p_{\text{Prior}}(\mathbf{s}) - \sigma R(\mathbf{s}))^2\right], \tag{1}$$

where $R(\mathbf{s})$ is the reward assigned to sequence $\mathbf{s}$ and σ is a scaling coefficient. Experience replay buffers can optionally be used to store high-scoring trajectories and reuse them during training, improving optimization stability.

Using the pretrained ChEMBL Prior (`data/Prior.ckpt`) and the standard REINVENT scoring utilities, we configured a Tanimoto-similarity–based reward. This choice follows a common use case of REINVENT, in which the generator is biased toward analogues of a small set of reference structures. Before reinforcement-learning-based optimization, we adapted the Prior network to the VDR ligand domain by transfer learning. All VDR ligand SMILES were canonicalized and subjected to basic filtering based on length and validity constraints. Starting from the ChEMBL-trained Prior, we fine-tuned the model by maximum-likelihood training using Adam optimization with an initial learning rate of 1×10^{-3}, periodic learning-rate decay by a factor of 0.97 every 500 steps, a batch size of 128, and a maximum SMILES length of 140 characters for 5 epochs. These hyperparameters were chosen to adapt the Prior gently to the VDR chemical space while limiting catastrophic forgetting of general chemical syntax and functional-group patterns.

Each generated string was validated with RDKit, and invalid or non-parsable SMILES were discarded. Across runs, the validity rate typically ranged between 60% and 80%, consistent with previous reports for REINVENT-like recurrent generators, while uniqueness among valid molecules remained high. We then computed standard physicochemical descriptors for the generated compounds and compared their distributions with those of the VDR training ligands. The close agreement in properties such as molecular weight, lipophilicity, and aromatic ring count indicates that transfer learning successfully adapted the Prior toward the VDR ligand domain.

Overall, REINVENT provided a flexible framework for *de novo* design in the focused VDR chemical space. At the same time, its output remained sensitive to the choice of reward function and to the alignment between the pretrained chemical prior and the target-domain ligand distribution. Transfer learning improved domain fit, but further improvements would likely require more expressive, multi-objective rewards that combine similarity, physicochemical constraints, and predicted activity.

1.1.2 Transmol

Transmol [3] is a transformer-based generative model that formulates molecular design as an autoregressive sequence-modeling problem over SMILES representations. By using multi-head self-attention [4], Transmol can capture long-range dependencies and syntactic regularities in SMILES strings that may be more difficult for purely recurrent architectures to model, while also enabling efficient parallel computation during training.

Input SMILES strings are tokenized at the character level, with each character mapped to an integer index and embedded into a continuous latent representation. The encoder

consists of a stack of transformer blocks, each containing multi-head self-attention, position-wise feed-forward layers, residual connections, and layer normalization. The decoder uses masked multi-head self-attention to enforce autoregressive generation, ensuring that predictions at each position depend only on previously generated tokens. Cross-attention layers allow the decoder to condition on the encoder output, and a final linear projection followed by log-softmax normalization produces token probability distributions at each decoding step.

The Transmol architecture used here consisted of 6 encoder layers and 6 decoder layers, with model dimensionality $d_{\text{model}} = 512$, feed-forward dimensionality $d_{\text{ff}} = 2048$, and 8 attention heads. Dropout rates between 0.1 and 0.3 were used to reduce overfitting.

We initialized Transmol from a checkpoint pretrained on the MOSES benchmark dataset [5], thereby leveraging prior knowledge of chemical syntax and broad structure–property relationships. All sequences were canonicalized and truncated to a maximum length of 100 characters, which was sufficient to accommodate the majority of benchmark ligands while minimizing unnecessary padding.

The model was fine-tuned using the Adam optimizer with momentum parameters $\beta_1 = 0.9$ and $\beta_2 = 0.98$, numerical stability constant $\epsilon = 10^{-9}$, and a scaling factor of 1.0. We used a batch size of 5,000 tokens to balance GPU memory constraints with effective gradient estimation, and applied label smoothing of 0.1 to the output distribution to improve generalization. Training was performed for up to 50 epochs, with early stopping based on validation loss using a patience of 5 epochs. This protocol was designed to preserve the pretrained model's general chemical knowledge while adapting it to the VDR ligand distribution.

The fine-tuned Transmol model was then used to generate focused libraries of VDR-like molecules by autoregressive greedy decoding, which favors high-probability sequences while maintaining tractable generation times. Generated sequences were validated with RDKit; invalid, truncated, or syntactically malformed SMILES were removed, and exact duplicates were eliminated during post-processing. The final number of valid unique molecules retained for downstream analysis is reported in Table S2.

The resulting Transmol library showed substantial structural diversity across a range of VDR-like chemotypes. Physicochemical descriptor and scaffold analyses indicated that Transmol transferred general chemical knowledge from pretraining to the more focused VDR domain, preserving chemical validity while exploring plausible molecular analogues beyond exact reproduction of the fine-tuning set.

1.1.3 GMDLDR

Generative Molecular Design in Low Data Regime (GMDLDR) [6] is a chemical language-modeling framework designed for settings in which training data are scarce. The approach combines recurrent neural networks with transfer learning to support *de novo* molecular design in focused chemical spaces. GMDLDR represents SMILES strings as sequences of discrete tokens and learns an autoregressive conditional probability distribution,

$$p(\mathbf{s}) = \prod_t p(s_t \mid s_{<t}). \tag{2}$$

Input SMILES strings are tokenized at the character level, converted to integer encodings, and projected through an embedding layer into continuous representations.

We followed the transfer-learning strategy described by **(author?)** [6]. The starting point was a pretrained chemical language model trained on a large ChEMBL24-derived

corpus, providing broad prior knowledge of drug-like chemical syntax and structure. For adaptation to the VDR domain, we froze the weights of the first LSTM layer, which contains 1,024 hidden units and captures general chemical syntax and patterns. We then fine-tuned the second LSTM layer with 256 hidden units, together with the embedding layer and output layer, on the VDR ligand dataset. Sequences were truncated to a maximum length of 140 characters to match the pretraining protocol.

Fine-tuning was performed using the Adam optimizer with a learning rate of 5×10^{-5} and learning-rate reduction on plateau, with a reduction factor of 0.5, patience of 3 epochs, and a minimum learning rate of 1×10^{-5}. We used a batch size of 16 and applied a dropout rate of 0.4 after each LSTM layer. An 80/20 molecular split into training and validation sets was used to monitor convergence and detect overfitting. Training was continued for 40 epochs, and model checkpoints were saved every 10 epochs.

1.1.4 Hyperparameters and implementation

Table S1 summarizes the hyperparameters employed in our VDR-focused GMDLDR fine-tuning.

Table S1: GMDLDR fine-tuning hyperparameters for the VDR ligand benchmark.

**Hyperparameter**	**Value**
SMILES Length Range	1–140
SMILES Augmentation	10-fold
First LSTM Hidden Units (frozen)	1024
Second LSTM Hidden Units (trainable)	256
Dropout Rate	0.4
Learning Rate	5×10^{-5}
Learning Rate Reduction Factor	0.5
Minimum Learning Rate	1×10^{-5}
Batch Size	16
Train/Validation Split	0.8 / 0.2
Epochs	40
Checkpoint Period	10

SMILES sequences were generated from the fine-tuned GMDLDR model at a sampling temperature of 0.7. Consistent with the other models, each sequence was validated using RDKit; control tokens (start ‘G’, end ‘E’, padding ‘A’) were removed, and invalid or non-parsable strings were discarded. The substantial majority of generated molecules proved chemically valid and unique with respect to both the training set and each other.

Structural analysis of the generated molecules revealed enrichment of known VDR-like scaffolds alongside the emergence of novel scaffolds absent from the fine-tuning set, indicating that the model generalizes beyond simple memorization. Physicochemical descriptor distributions remained closely aligned with those of the VDR ligands, consistent with the low-data design of GMDLDR.

Table S2: Number of generated molecules, the number of recreated reference molecules and the recreation rate for the three considered generative models, fine-tuned to VDR binders.

**Model**	**Generated**	**Recreated**	**Rate**
REINVENT	7,816	25 (6.8%)	3.2×10^{-3}
GMDLDR	6,962	21 (5.7%)	3.0×10^{-3}
Transmol	1,134	6 (1.6%)	5.3×10^{-3}

1.2 Evaluation techniques for generative output

1.2.1 Recreation rate of reference molecules

Table S2 summarizes the recreation rate of reference molecules for the three generative models. *Generated* denotes the total number of molecules produced by each model, *Recreated* denotes the number of unique reference ligands reproduced by the generator, with the percentage relative to the 503-ligand reference set shown in parentheses, and *Rate* quantifies sampling efficiency as the ratio of recreated reference ligands to generated molecules. REINVENT and GMDLDR achieved the highest recreation coverage, reproducing 6.8% and 5.7% of the reference ligands, respectively, indicating that these models more frequently sampled structures close to the known VDR ligand distribution. Transmol recreated fewer reference ligands overall, 1.6%, but showed comparable sampling efficiency given its smaller generated library. Overall, the recreation rate quantifies overlap with known actives and should not be interpreted as a direct proxy for binding strength. We therefore evaluated generated candidates using orthogonal predictors, including DTA models, docking, and Boltz-2-derived metrics, and relied on the multimodal fusion framework for final prioritization.

1.2.2 Overlap of generative models

To assess whether different generative models captured shared chemical patterns from known VDR binders, we analyzed the overlap among their generated chemical spaces. Each generative model was fine-tuned on the same set of experimentally validated VDR ligands and then used to generate candidate molecules. By comparing molecules independently generated by multiple models with those produced by only one model, we aimed to distinguish robust chemical patterns consistently recovered across architectures from model-specific biases or divergent exploration of chemical space.

The rationale behind this analysis is that molecules independently generated by multiple architectures may reflect chemical motifs that are strongly represented in the VDR ligand distribution, whereas molecules unique to a single model may represent either broader chemical exploration or architecture-specific generation patterns. To quantify these relationships systematically, we performed pairwise comparisons of the generated libraries using canonical SMILES representations.

Based on this overlap analysis, we partitioned the generated chemical space into distinct groups, referred to as G-groups, defined by the number of generative models that independently produced each molecule. Specifically, G1 comprises molecules generated by exactly one model and not reproduced by any other model in the ensemble. These molecules may reflect either novel exploration of chemical space or model-specific generation behavior.

G2 consists of molecules generated by exactly two models, indicating intermediate cross-model consensus. G3 consists of molecules generated by all three models, representing the strongest consensus class and potentially capturing chemical features consistently learned across the three architectures.

This grouping strategy enables several analyses of generative model behavior and chemical-space exploration. First, by comparing structural features and predicted binding profiles across G-groups, we can assess whether high-consensus molecules, particularly those in G3, show stronger VDR-like properties than model-specific outputs in G1. Second, the chemical diversity within each group can be examined to determine whether multiple models converge on similar scaffolds or instead recover complementary regions of chemical space. Third, the distribution of molecules across G-groups provides a quantitative measure of agreement among the generative approaches and helps identify which model combinations contribute most strongly to shared candidate generation.

1.2.3 DTA models

Drug–target affinity (DTA) predictors estimate binding-related quantities by learning statistical patterns from experimental affinity data, whereas docking-based methods approximate ligand–protein interactions through structure-based scoring functions. These two classes of methods therefore capture different aspects of ligand–target recognition and need not produce concordant rankings. In the present study, we used DTA predictions as complementary evidence for prioritizing generated VDR candidate molecules.

Binding affinity is a central property of protein–ligand interaction and is widely used as a ranking criterion in virtual screening. We computed DTA predictions between the generated molecular candidates and the VDR target using GraphDTA [7] and a multitask label-encoding model (MLT-LE). For GraphDTA, we used three model variants from the GraphDTA framework, namely GAT-GCN, GCNNet, and GINConvNet, trained separately for different affinity endpoints. These models represent small molecules as molecular graphs and encode protein targets using one-dimensional convolutional neural networks. The GraphDTA models were implemented using PyTorch Geometric [8]. In addition, we used MLT-LE, a multitask affinity-prediction model that jointly learns from multiple binding-affinity endpoints, including K_{D}, K_{I}, IC_{50}, and EC_{50}.

The training datasets for GraphDTA and MLT-LE were obtained from TDC BindingDB and BindingDB v2022m3, respectively, both derived from BindingDB [9]. Only human target-specific records were retained. Because both datasets contain multiple affinity measurements for the same drug–target pairs from different experimental assays, duplicate drug–target records were aggregated by taking the median affinity value. For the TDC BindingDB data, records with affinity variance greater than two standard deviations for the same drug–target pair were removed before aggregation. Invalid SMILES strings in BindingDB v2022m3 were removed and the remaining molecules were canonicalized using RDKit [10].

After preprocessing, the TDC BindingDB dataset contained 42,236, 296,685, and 766,904 unique drug–target pairs for K_{D}, K_{I}, and IC_{50}, respectively, with no EC_{50} endpoint used for GraphDTA training. BindingDB v2022m3 contained 39,379, 214,052, 610,502, and 84,487 unique drug–target pairs for K_{D}, K_{I}, IC_{50}, and EC_{50}, respectively. Three GraphDTA models were trained separately on the TDC BindingDB K_{D}, K_{I}, and IC_{50} subsets, whereas a single MLT-LE model was trained jointly on all four BindingDB v2022m3 affinity endpoints. The resulting target-general DTA predictors were then applied

to the generated molecules and VDR target sequence to obtain affinity-based ranking signals for multimodal fusion.

1.2.4 AutoDock Vina

We used AutoDock Vina [11], a widely used open-source molecular docking program, to estimate ligand–receptor binding poses and docking scores. AutoDock Vina employs an empirical scoring function that combines steric interactions, hydrogen bonding, hydrophobic effects, and conformational terms to efficiently approximate protein–ligand binding energies.

For receptor preparation, we obtained the crystal structure of the vitamin D receptor ligand-binding domain in complex with 1α,25-dihydroxyvitamin D_3 from the Protein Data Bank (PDB ID: 1DB1; resolution: 1.80 Å). The receptor structure was prepared by removing water molecules and adding hydrogen atoms at physiological pH 7.4 using AutoDockTools 1.5.7 [12]. Gasteiger partial charges were assigned to all atoms, and the receptor was converted to PDBQT format, which encodes the atomic coordinates, partial charges, and atom types required by AutoDock Vina.

Ligand preparation began with conversion of SMILES strings into three-dimensional conformers using RDKit [10]. For each generated molecule, the initial 3D conformer was energy-minimized using the Merck Molecular Force Field 94 (MMFF94) [13]. Rotatable bonds were automatically detected, and ligands were converted to PDBQT format while preserving the corresponding torsional degrees of freedom for conformational sampling during docking.

The docking search space was defined as a cubic grid box centered on the centroid of the reference ligand, with coordinates $x = 15.2$ Å, $y = 42.1$ Å, and $z = 35.8$ Å. The grid dimensions were set to $25 \times 25 \times 25$ Å, ensuring coverage of the ligand-binding pocket and surrounding residues. Docking calculations were performed with an exhaustiveness parameter of 8, generating up to 10 binding modes within an energy range of 3 kcal/mol from the best-scoring pose. The exhaustiveness parameter controls the thoroughness of the global search, with higher values increasing computational cost but improving conformational sampling.

AutoDock Vina reports docking scores in kcal/mol, where more negative values indicate stronger predicted binding. Within the CWRA framework, Vina scores were direction-corrected so that larger values consistently indicate stronger binding evidence, and were then normalized to the interval $[0, 1]$ using per-modality min–max normalization over the evaluated molecular pool, as described in the main text.

1.2.5 Boltz-2 structure prediction and scoring

Unlike traditional physics-based docking methods, Boltz-2 uses a generative diffusion framework for protein–ligand complex prediction. The model is trained on non-redundant protein structures from the Protein Data Bank, filtered at 70% sequence identity to promote structural diversity and reduce bias toward overrepresented protein families.

Boltz-2 jointly models protein and ligand coordinates in three-dimensional space, iteratively refining initial configurations toward physically plausible protein–ligand complexes. In our workflow, the model was provided with the amino acid sequence of the vitamin D receptor ligand-binding domain and the SMILES representation of each candidate ligand, and was used to predict the atomic coordinates of the bound complex. Compared with rigid or semi-flexible docking protocols, this approach can in principle account for

ligand-dependent structural rearrangements and induced-fit effects, although such predictions should still be interpreted as model-derived hypotheses rather than experimentally resolved binding modes.

For each predicted complex, Boltz-2 provides two complementary outputs. First, it returns a model-derived binding-affinity score, reported on a scale analogous to structure-based scoring functions. Second, it provides per-atom confidence estimates analogous to pLDDT-like confidence scores, reflecting the model's certainty in the predicted atomic positions. We computed the average confidence score across all ligand heavy atoms to obtain an overall ligand-level confidence measure ranging from 0 to 100, where higher values indicate greater confidence in the predicted ligand placement.

Both the Boltz-2 affinity and ligand-confidence metrics were included as separate modalities in the CWRA framework. Scores were direction-corrected where appropriate and then normalized to the interval $[0, 1]$ using per-modality min–max normalization over the evaluated molecular pool, as described in the main text. This preprocessing placed Boltz-2-derived signals on a common scale with the other structure-based and machine-learning-based modalities, allowing them to contribute directly to the weighted ensemble ranking.

2 Results

2.1 GraphDTA and MLT-LE

Table S3 summarizes the distribution of predicted affinities across the full evaluated ligand set for each unimodal predictor. MLT-LE produced pK_d values with a median of 8.12 (IQR 6.83–9.18). The GraphDTA/GCNNet predictors yielded median values of 6.70 for K_d (IQR 5.90–7.80), 8.66 for K_i (IQR 8.36–8.91), and 5.58 for IC_{50} (IQR 5.03–6.27). GraphDTA outputs are log-transformed affinity values (negative $\log_{10}$ of binding constants). For these log-transformed affinity endpoints, higher values indicate stronger predicted binding.

Table S3: Summary statistics of computational predictions for VDR ligands across the evaluated candidate set (N = 16,196).

**Modality**	**Median**	**IQR**	**Range**
GraphDTA pK_d	6.70	(5.90, 7.80)	(4.06, 9.36)
GraphDTA pK_i	8.66	(8.36, 8.91)	(5.05, 9.83)
GraphDTA pIC_{50}	5.58	(5.03, 6.27)	(4.04, 8.00)
MLT-LE pK_d	8.12	(6.83, 9.18)	(2.97, 9.93)
AutoDock Vina[a]	−10.10	(−11.20, −8.90)	(−13.80, −1.50)
Boltz-2 Affinity	−0.30	(−1.04, 0.30)	(−4.49, 2.50)
Boltz-2 Confidence	0.58	(0.57, 0.59)	(0.50, 0.94)
DrugBAN	−8.14	(−8.44, −7.82)	(−9.79, −6.40)
MolTrans	−7.58	(−7.79, −7.37)	(−8.75, −6.25)
TankBind	−9.05	(−9.36, −8.74)	(−10.49, −7.35)
Uni-Mol Similarity	0.14	(0.09, 0.40)	(0.01, 0.98)

2.2 TankBind affinity

TankBind predicted binding affinities showed a narrow distribution with a median of −9.05 kcal/mol (IQR: −9.36 to −8.74; range: −10.49 to −7.35). This compressed dynamic range suggests that TankBind's predictions for this target and chemical series are relatively uniform, potentially reflecting limited discriminative power among the VDR candidate pool. The low variance may arise from the geometric similarity of candidates to the VDR pocket or from model characteristics when applied to a focused chemical library.

2.3 DrugBAN affinity

DrugBAN predicted affinities spanned a broader range than TankBind, with a median of −8.14 kcal/mol (IQR: −8.44 to −7.82; range: −9.79 to −6.40). DrugBAN's bilinear attention mechanism models atom–residue interactions from molecular graphs and protein sequences, providing a ranking signal that was only weakly concordant with several other modalities (CI = 0.45–0.60). Reference compounds showed a slightly stronger median predicted affinity than generated candidates (−8.31 versus −8.13 kcal/mol), although the overall separation between these groups was modest.

2.4 MolTrans affinity

MolTrans predicted affinities showed a median of −7.58 kcal/mol (IQR: −7.79 to −7.37; range: −8.75 to −6.25). As a transformer-based architecture designed to capture long-range sequence dependencies, MolTrans provides a ranking signal that is distinct from graph-based and docking-based modalities. Its low concordance with other predictors (CI = 0.46–0.57) further supports its role as a complementary modality within the CWRA ensemble.

2.5 AutoDock Vina

Molecular docking with AutoDock Vina was used to assess the binding potential of all generated compounds against the VDR ligand-binding domain (PDB ID: 1DB1). Predicted binding affinities spanned an approximately 15 kcal/mol range, from about −1 to −15.5 kcal/mol, indicating substantial variation in docking scores across the generated library. Calcitriol, the natural VDR ligand, scored −12.6 kcal/mol, placing it among the top-scoring compounds and supporting the plausibility of the docking setup.

Using calcitriol as a reference point, G2 compounds and reference ligands showed the most favorable median Vina scores (−11.2 and −11.0 kcal/mol, respectively), followed by G3 compounds (−10.5 kcal/mol) and G1 compounds (−10.1 kcal/mol). Comparison of Vina scores with ML-based predictors, including MLT-LE and GraphDTA endpoints, revealed weak correlations ($|r| = 0.13$–0.25), suggesting limited concordance between physics-based and data-driven scoring modalities. This limited agreement underscores their complementarity: Vina captures geometric and energetic fit within the binding pocket, whereas ML models learn statistical patterns from large-scale binding data that may include factors beyond local structural compatibility.

2.6 Boltz-2

Boltz-2 provided two outputs for each candidate: a predicted binding-affinity score and a confidence score reflecting the reliability of the predicted protein–ligand complex structure. Across the evaluated dataset, Boltz-2 confidence scores ranged from 0.501 to 0.940. The mean Boltz-2 affinity score was -0.39, while calcitriol achieved a stronger score of -2.19 and a high confidence score of 0.932. The correlation between Vina scores and Boltz-2 affinity values was very weak ($r = 0.06$), indicating that the two structure-based methods provide largely distinct ranking signals despite both being based on protein–ligand structural information.

The Boltz-2 confidence score, with a median of 0.581 and a narrow interquartile range, showed limited variation across compounds. This tight distribution suggests that predicted structural confidence was relatively uniform across the evaluated ligands and was not simply determined by predicted affinity. Confidence scores also showed weak concordance with other modalities, with CI values ranging from 0.38 to 0.62, indicating that predicted structural reliability and predicted binding strength capture partly orthogonal aspects of ligand quality.

We observed a weak positive trend between MLT-LE pK_d values and Boltz-2 confidence, with higher predicted affinity corresponding to marginally higher confidence scores. This may reflect better structural complementarity for some high-scoring compounds, although the effect should be interpreted cautiously. Consistent with the Vina results, G1 and G2 compounds achieved stronger Boltz-2 affinity scores than G3 compounds, supporting the use of multimodal fusion rather than relying on any single structure-based predictor.

2.7 Uni-Mol similarity

Uni-Mol similarity scores were computed in Uni-Mol embedding space as the cosine similarity of each candidate's embedding to a reference centroid derived from known VDR binders (as described in the main text). During repeated split evaluation, the centroid is recomputed using training-split actives only (split-honest computation). The resulting similarity scores showed a median of 0.138 (IQR: 0.092–0.398; range: 0.013–0.980).

2.8 Non-steroidal candidates

Figure S1 shows the ten highest-scoring non-steroidal candidates.

2.9 Full-pool min–max normalization analysis

To assess potential leakage through full-pool min–max normalization, we repeated the VDR cross-validation using strict fold-isolated normalization. In this setting, held-out test actives were excluded from estimation of normalization parameters in each fold, and the resulting parameters were then applied to the corresponding test fold. Performance was essentially unchanged: EF@1% remained identical between the default and strict settings (default: 21.32 ± 6.41; strict: 21.32 ± 5.67), and differences in other metrics were small and within one standard deviation (ΔAUROC = +0.009, ΔBEDROC = +0.002, ΔEF@5% = +0.21). These results indicate that the reported enrichment estimates are not driven by normalization leakage.

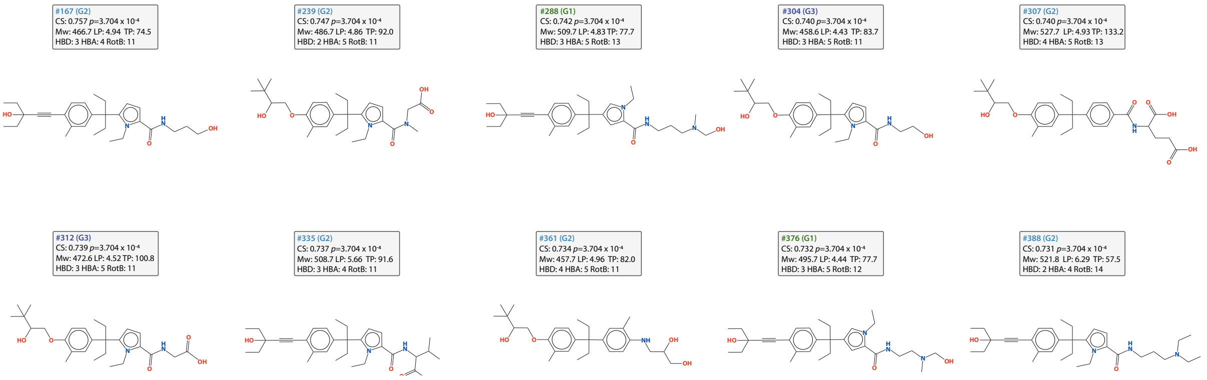

Figure S1: **Representative highest-scoring non-steroidal CWRA-selected candidates.** Structures are shown together with CWRA score (CS), conformal p-value, generator-overlap class (G1–G3), and selected physicochemical descriptors. These examples illustrate the non-steroidal component of the CWRA-selected chemical space and are not intended to imply experimental validation.

2.10 CWRA Weight Sensitivity Analysis

To assess whether the CWRA ranking is sensitive to precise weight values, we perturbed the mean weights obtained from the five repeated random splits by additive uniformly distributed noise, $\delta_m \in [-\Delta, +\Delta]$, independently for each modality weight w_m. The perturbed weights were then re-projected onto the capped simplex to preserve the CWRA constraints, i.e. non-negativity, $\sum_m w_m = 1$, and the predefined lower and upper weight bounds. For each perturbation level, we generated 200 perturbed weight vectors and recomputed the CWRA ranking. Ranking stability was quantified by overlap with the baseline ranking at Top-100, Top-500, and Top-1000, and by Spearman rank correlation over the Top-2000. Results are shown in Table S4.

Table S4: CWRA ranking stability under weight perturbation (200 trials per Δ, mean $\pm$ SD).

**Perturbation**	**Overlap [%]**			**Spearman ρ**
$\pm\Delta$	Top-100	Top-500	Top-1000	Top-2000
±1%	97 ± 1	98 ± 1	99 ± 0	0.998 ± 0.002
±5%	89 ± 4	94 ± 2	97 ± 1	0.971 ± 0.024
±10%	82 ± 5	88 ± 4	91 ± 5	0.907 ± 0.082
±20%	73 ± 9	79 ± 8	81 ± 13	0.785 ± 0.169
±50%	60 ± 16	68 ± 12	71 ± 18	0.658 ± 0.226

The CWRA ranking was highly stable under small additive perturbations of the learned weights. At ±1% perturbation, 97±1% of the Top-100 compounds, 98±1% of the Top-500 compounds, and 99 ± 0% of the Top-1000 compounds were retained, with Spearman $\rho = 0.998 \pm 0.002$ over the Top-2000. Under the larger ±5% perturbation, Top-100 retention remained high (89 ± 4%), and rank correlation remained strong ($\rho = 0.971 \pm 0.024$). Even at ±10%, which represents a substantial absolute perturbation relative to several learned modality weights, 82 ± 5% of the Top-100 compounds and 91 ± 5% of the Top-1000 compounds were retained. As expected, larger perturbations led to gradual degradation in ranking stability, but even the extreme ±50% setting retained 60 ± 16% of the Top-100

compounds.

These results indicate that CWRA prioritization is robust to small-to-moderate deviations from the optimized weight vector and does not depend on finely tuned numerical weights. The observed stability is consistent with the enrichment of cross-generator consensus compounds in the Top-100 (see Section 2.12 below), including G2 compounds (32/100; 29.0× enrichment) and G3 compounds (12/100; 59.6× enrichment), whose rankings are supported by convergent multimodal evidence rather than by a single precisely weighted modality.

2.11 Effect of modality weight bounds

To assess whether CWRA performance depends critically on the imposed modality-weight bounds, we repeated the VDR repeated-split evaluation under three bound configurations: loose bounds ($[0.01, 0.50]$), the default bounds ($[0.03, 0.25]$), and tighter bounds ($[0.05, 0.20]$). Performance was stable across settings, with EF@1% values remaining within one standard deviation of the default result (see Table S5). The Top-100 overlap with the default CWRA ranking was also high for both alternative bound settings, indicating that the prioritized candidates are not strongly driven by the exact numerical choice of bounds.

Table S5: Sensitivity of CWRA performance to modality-weight bounds. EF@1% is reported as mean ± SD across repeated splits. Top-100 overlap is computed relative to the default CWRA ranking.

**Bounds**	**EF@1% (mean±SD)**	**Top-100 overlap**
$[0.01, 0.50]$ (loose)	20.87 ± 6.10	94%
$[0.03, 0.25]$ (default)	21.32 ± 6.41	—
$[0.05, 0.20]$ (tight)	20.14 ± 5.88	91%
Equal weights (baseline)	10.79 ± 2.81	78%

2.12 G-Group Enrichment in the CWRA Top-100

A central premise of the CWRA pipeline is that cross-generator consensus may provide an additional prioritization signal: molecules generated independently by multiple architectures are less likely to reflect idiosyncratic sampling behavior of a single model. We therefore examined the representation of G1, G2, and G3 compounds in the CWRA Top-100 relative to their frequencies in the generated pool. Table S6 summarizes the results.

Table S6: G-group representation in the CWRA Top-100 relative to the generated pool. Enrichment is defined as the fraction in the Top-100 divided by the corresponding pool fraction.

**G-Group**	**Set size**	**Pool rate**	**In Top-100**	**Enrichment**	**CWRA score**
G1	14,211	98.7%	56	0.6×	0.523 ± 0.074
G2	159	1.1%	32	29.0×	0.709 ± 0.062
G3	29	0.2%	12	59.6×	0.745 ± 0.040

G3 compounds, produced independently by all three generator architectures, are strongly enriched in the CWRA Top-100 relative to their pool representation (0.2% of the pool versus 12% of the Top-100; 59.6× enrichment). G2 compounds are similarly enriched (29×), whereas G1-only compounds are depleted (0.6×). The progressive increase in CWRA meta-score from G1 (0.523 ± 0.074) through G2 (0.709 ± 0.062) to G3 (0.745 ± 0.040) indicates that cross-generator consensus correlates with stronger multimodal scoring. This does not by itself validate binding activity, but it provides an additional, model-architecture-level prioritization signal that is orthogonal to the individual scoring modalities.

2.13 Internal Chemical Diversity of the Generated Library

To quantify chemical diversity beyond scaffold counts, we computed internal diversity using ECFP4 fingerprints. For each compound group, pairwise Tanimoto similarities were computed from RDKit Morgan fingerprints, and internal diversity was defined as the mean pairwise Tanimoto distance, ($1 - \text{Tanimoto}$). Higher values therefore indicate greater chemical diversity within the group.

The generated pool showed high internal diversity, with a mean pairwise Tanimoto distance of 0.86 (Table S7). G1 compounds showed the same internal diversity (0.86), consistent with broad single-generator exploration and the numerical dominance of G1 compounds in the generated pool. In contrast, G2 and G3 compounds showed progressively lower internal diversity (0.68 and 0.66, respectively), consistent with convergence of independent generators on more closely related chemotypes. Thus, the generated library combines broad overall chemical diversity with a smaller cross-generator consensus region enriched for related VDR-compatible chemotypes.

Table S7: Internal chemical diversity of generated compounds. Diversity is measured as mean pairwise Tanimoto distance using ECFP4 fingerprints.

**Group**	**Mean pairwise Tanimoto distance**
Generated pool	0.86
G1	0.86
G2	0.68
G3	0.66

2.14 Generative Model Physicochemical Evaluation

To assess whether generated and CWRA-prioritized compounds occupy a physicochemical regime comparable to known VDR ligands, we compared standard RDKit-derived descriptors across reference actives, generated G-groups, and the CWRA Top-100 (Table S8).

The CWRA Top-100 closely matches the reference distribution in molecular weight, cLogP, tPSA, and QED, indicating that prioritization does not shift the selected candidates into a markedly different physicochemical regime. The slightly elevated FractionCSP3 (0.70 ± 0.13) and stereocentre count (5.5 ± 2.8) in the Top-100 relative to the full G1 pool reflect CWRA's preference for three-dimensionally complex, secosteroid-like compounds. The SA Score of the Top-100 (4.50 ± 0.77) indicates moderate synthetic complexity, comparable to the reference VDR ligands (4.21 ± 0.90) and consistent with the structural

Table S8: Physicochemical properties by compound group (mean ± SD).

Property	Reference	G1	G2	G3	Top-100
MW (Da)	464.6 ± 66.2	441.1 ± 124.8	480.4 ± 89.3	477.3 ± 49.0	451.0 ± 47.4
cLogP	5.64 ± 1.37	5.18 ± 2.42	5.94 ± 1.54	5.84 ± 0.82	5.76 ± 0.90
tPSA (Å^2)	69.7 ± 20.8	67.7 ± 28.5	73.4 ± 23.1	72.8 ± 16.1	67.9 ± 12.8
QED	0.39 ± 0.15	0.43 ± 0.17	0.38 ± 0.14	0.40 ± 0.11	0.42 ± 0.11
SA Score	4.21 ± 0.90	4.11 ± 1.08	4.42 ± 0.91	4.57 ± 0.73	4.50 ± 0.77
FractionCSP3	0.65 ± 0.16	0.65 ± 0.21	0.64 ± 0.18	0.66 ± 0.13	0.70 ± 0.13
Stereocenters	4.4 ± 3.1	3.7 ± 3.1	4.6 ± 2.7	5.2 ± 2.4	5.5 ± 2.8

complexity of secosteroidal VDR chemotypes. These values should therefore be interpreted as supporting chemical plausibility rather than guaranteeing straightforward synthesis.

2.15 Generalization to an additional target: $GABA_A$

To probe whether CWRA generalizes beyond the VDR case study, we repeated the full pipeline on an additional target, the $GABA_A$ receptor, using curated experimental actives and *de novo* generated candidate pools. Active compounds were assembled from *ChEMBL*, *PubChem*, and *ZINC* [2, 14, 15], standardized using the same RDKit pipeline, and used to fine-tune the generative models and construct target-specific candidate libraries analogously to the VDR workflow. The same eleven scoring modalities, drug-likeness filtering, repeated held-out-active splitting protocol, and split-honest Uni-Mol centroid computation were then applied as described for VDR.

On $GABA_A$, target-specific CWRA achieved the strongest very-early enrichment among all evaluated fusion and individual-modality baselines (Table S9). CWRA reached EF@1% = 25.41 ± 3.96, compared with 17.47 ± 6.17 for equal-weight fusion and 11.64 ± 2.12 for the best individual modality at this cutoff (Boltz-2 confidence). CWRA also achieved the strongest performance at 2.5% and 5% screening depths. At broader cutoffs, AutoDock Vina became strongest, consistent with CWRA being optimized primarily for very-early enrichment rather than uniform dominance across all screening depths.

2.15.1 Cross-target source-model transfer

The learned CWRA weights differed substantially between VDR and $GABA_A$ (Table S10), indicating that the most informative modalities are target-dependent. To assess whether a complete CWRA model trained on one target transfers to the other without recalibration, we performed a strict cross-target source-model transfer experiment (Table S11).

Cross-target transfer was evaluated by applying the five CWRA models learned on the source target directly to the destination dataset. For each source fold, modality scores were normalized using source-dataset ranges, source-specific preprocessing was retained, and Uni-Mol similarity was calculated against a centroid constructed only from that fold's source training actives. The resulting source-fold weights were then applied without re-optimization to the destination compounds, and the five transferred scores were averaged to produce a final ranking. Destination labels were used only to calculate enrichment on held-out active splits and did not influence normalization, Uni-Mol centroids, weights, or ranking.

Table S9: Extended virtual screening performance of individual scoring methods and fusion baselines on the $GABA_A$ ligand discovery task (mean ± std). N=12,571 compounds (252 actives); 5 repeated random splits of actives (train fraction = 0.85); split seed=42; drug-likeness pre-filter removed 45/12,616 compounds; top-k cutoffs: @1%=125, @2.5%=314, @5%=628, @10%=1257, @20%=2514. EF: Enrichment Factor; Hits: number of held-out active compounds retrieved. **Bold**: best; underlined: second best.

	Enrichment Factor (EF)					Hits				
Method	@1%	@2.5%	@5%	@10%	@20%	@1%	@2.5%	@5%	@10%	@20%
GraphDTA K_d	0.53±1.06	1.90±1.23	2.00±0.97	1.37±0.39	1.08±0.26	0±0	2±1	4±2	5±1	8±2
GraphDTA K_i	6.88±3.59	4.42±1.69	5.69±1.39	4.05±0.54	2.87±0.30	3±1	4±2	11±3	15±2	22±2
GraphDTA IC_{50}	0.00±0.00	0.21±0.42	0.42±0.39	0.47±0.31	0.95±0.21	0±0	0±0	1±1	2±1	7±2
MLT-LE pK_d	0.53±1.06	0.21±0.42	0.32±0.42	0.26±0.29	0.97±0.20	0±0	0±0	1±1	1±1	7±1
TankBind	0.00±0.00	0.00±0.00	0.00±0.00	0.16±0.21	0.61±0.16	0±0	0±0	0±0	1±1	5±1
DrugBAN	0.00±0.00	0.63±0.52	1.26±0.42	1.37±0.59	1.45±0.19	0±0	1±0	2±1	5±2	11±1
MolTrans	2.65±2.37	1.90±1.93	1.37±0.92	1.16±0.76	1.53±0.27	1±1	2±2	3±2	4±3	12±2
AutoDock Vina	1.59±1.30	5.27±2.21	9.48±1.45	**9.32±0.27**	**5.00±0.00**	1±0	5±2	18±3	**35±1**	**38±0**
Boltz-2 affinity	0.00±0.00	0.00±0.00	0.74±0.26	1.00±0.26	0.74±0.38	0±0	0±0	1±0	4±1	6±3
Boltz-2 confidence	11.64±2.12	10.32±2.04	9.69±0.98	8.84±0.46	4.42±0.23	4±1	10±2	18±2	34±2	34±2
Uni-Mol	7.94±2.90	6.53±1.03	4.64±0.61	3.95±0.62	3.05±0.49	3±1	6±1	9±1	15±2	23±4
Equal-weight	17.47±6.17	12.64±1.76	10.22±1.78	6.74±0.77	4.11±0.15	7±2	12±2	19±3	26±3	31±1
Random-weight fusion	11.94±2.80	9.61±1.55	7.42±0.75	5.13±0.27	3.30±0.08	4±1	9±1	14±1	19±1	24±1
CWRA	**25.41±3.96**	**22.12±3.46**	**14.33±0.84**	8.47±0.31	4.68±0.11	**10±1**	**21±3**	**27±2**	32±1	36±1

Strict source-model transfer produced above-random enrichment but was substantially weaker than target-specific CWRA. VDR-derived models transferred to $GABA_A$ achieved EF@1% $= 9.53 \pm 3.96$, compared with 25.41 ± 3.96 for target-specific $GABA_A$ CWRA. Conversely, $GABA_A$-derived models transferred to VDR achieved EF@1% $= 9.74 \pm 1.34$, compared with 21.32 ± 6.41 for target-specific VDR CWRA. These results indicate that CWRA generalizes as a target-adaptive calibration framework, while the learned weights, normalization parameters, and ligand-centroid similarity components remain target-specific and should not be assumed to transfer unchanged across targets.

Table S10: Average CWRA modality weights for VDR and $GABA_A$ across cross-validation splits (mean ± SD; VDR $n = 5$, $GABA_A$ $n = 5$). Absolute change is calculated as $w_{\text{GABA}_A} - w_{\text{VDR}}$.

Modality	VDR weight	$GABA_A$ weight	Absolute change
GraphDTA K_d	0.037±0.008	0.030±0.000	-0.007
GraphDTA K_i	0.030±0.001	0.103±0.017	+0.073
GraphDTA IC_{50}	0.242±0.002	0.031±0.002	-0.211
MLT-LE pK_d	0.030±0.000	0.030±0.000	+0.000
TankBind	0.069±0.041	0.036±0.013	-0.034
DrugBAN	0.101±0.014	0.189±0.014	+0.089
MolTrans	0.042±0.010	0.061±0.024	+0.019
AutoDock Vina	0.206±0.036	0.184±0.020	-0.022
Boltz-2 affinity	0.053±0.025	0.050±0.019	-0.003
Boltz-2 confidence	0.155±0.044	0.083±0.012	-0.072
Uni-Mol	0.034±0.004	0.203±0.012	+0.169

Table S11: Early-enrichment performance of target-specific CWRA, equal-weight fusion, the best fixed individual modality, and cross-target weight transfer. All results are mean ± SD over five held-out test splits. Cross-target transfer uses source weights, source normalization, and source Uni-Mol centroids.

Target	Method	EF@1%	EF@2.5%	EF@5%
VDR	CWRA	21.32±6.41	14.13±2.34	9.47±0.60
VDR	Equal-weight	10.79±2.81	7.80±1.22	6.32±1.30
VDR	Best modality (GraphDTA IC_{50})	16.32±3.96	12.23±1.35	8.32±0.76
VDR	$GABA_A$ weights → VDR	9.74±1.34	8.75±0.63	6.26±1.08
$GABA_A$	CWRA	25.41±3.96	22.12±3.46	14.33±0.84
$GABA_A$	Equal-weight	17.47±6.17	12.64±1.76	10.22±1.78
$GABA_A$	Best modality (Boltz-2 confidence)	11.64±2.12	10.32±2.04	9.69±0.98
$GABA_A$	VDR weights → $GABA_A$	9.53±3.96	8.01±1.58	6.74±1.35

2.16 Statistical Analysis

For each target, performance was evaluated using 100 repeated random holdout splits of the known active compounds, with 85% of the actives used for weight optimization and 15% retained for testing. The unlabeled/background compounds remained in the ranked screening library for every split. CWRA weights were learned using only the training actives, and Uni-Mol similarity was recomputed independently within each split using only those training actives. EF values at 1%, 2.5%, 5%, 10%, and 20% were then calculated for the held-out actives. CWRA was compared with equal-weight fusion and with a pre-specified best individual modality at the primary EF@1% cutoff: GraphDTA (IC_{50}) for VDR and Boltz-2 confidence for $GABA_A$. All comparisons were paired because CWRA and its comparator were evaluated on identical splits. Because this statistical analysis used 100 repeated splits, the mean EF values differ from the five-split summary tables reported elsewhere.

Statistical significance was assessed using one-sided Nadeau–Bengio corrected resampled (t)-tests, with the pre-specified superiority alternative that CWRA produced a higher EF than the comparator. For each comparison, the split-wise EF difference was calculated as ($\mathrm{EF}_{\mathrm{CWRA}} - \mathrm{EF}_{\mathrm{comparator}}$). The standard error was corrected for overlap between repeated training and test sets using ($\sqrt{(1/n + n_{\mathrm{test}}/n_{\mathrm{train}})s^2}$), where (n=100) and ($n_{\mathrm{test}}/n_{\mathrm{train}} = 0.15/0.85$), corresponding to the active-compound holdout ratio. Two-sided 95% confidence intervals are reported descriptively for the mean EF difference. Holm correction was applied separately within each target across the five cutoffs and two comparators, giving ten simultaneous tests per target. Statistical significance was defined as a Holm-adjusted ($p < 0.05$). Results are reported in Table S12.

Table S12: Paired comparison of test-set enrichment factors across 100 repeated random splits for VDR and $GABA_A$. The pre-specified individual-modality comparators were the strongest single modalities at the primary EF@1% cutoff: GraphDTA IC_{50} for VDR and Boltz-2 confidence for $GABA_A$. The reported p-values are from one-sided Nadeau–Bengio corrected resampled t-tests for the superiority alternative that CWRA has higher EF, with Holm adjustment across the five cutoffs and two comparators within each target. Confidence intervals are unadjusted two-sided 95% intervals for the mean paired EF difference.

Target	Cutoff	Comparator	n	CWRA EF	Comparator EF	Δ EF [95% CI]	p_{Holm}	Significant
VDR	@1%	GraphDTA IC_{50}	100	22.11	19.17	2.93 [0.01, 5.86]	0.048	Yes
VDR	@1%	Equal-weight	100	22.11	11.99	10.12 [6.52, 13.72]	4.15×10^{-7}	Yes
VDR	@2.5%	GraphDTA IC_{50}	100	14.55	13.29	1.26 [0.01, 2.51]	0.048	Yes
VDR	@2.5%	Equal-weight	100	14.55	8.49	6.07 [4.62, 7.52]	1.70×10^{-12}	Yes
VDR	@5%	GraphDTA IC_{50}	100	9.72	8.34	1.38 [0.86, 1.91]	1.21×10^{-6}	Yes
VDR	@5%	Equal-weight	100	9.72	6.49	3.23 [2.54, 3.92]	1.38×10^{-14}	Yes
VDR	@10%	GraphDTA IC_{50}	100	7.37	4.72	2.65 [2.24, 3.06]	2.25×10^{-22}	Yes
VDR	@10%	Equal-weight	100	7.37	5.07	2.30 [1.90, 2.70]	6.44×10^{-19}	Yes
VDR	@20%	GraphDTA IC_{50}	100	4.62	2.73	1.89 [1.54, 2.23]	6.61×10^{-18}	Yes
VDR	@20%	Equal-weight	100	4.62	3.14	1.47 [1.11, 1.84]	5.01×10^{-12}	Yes
$GABA_A$	@1%	Boltz-2 confidence	100	25.80	11.51	14.29 [8.47, 20.11]	8.37×10^{-6}	Yes
$GABA_A$	@1%	Equal-weight	100	25.80	18.16	7.65 [3.25, 12.04]	0.001	Yes
$GABA_A$	@2.5%	Boltz-2 confidence	100	21.14	10.49	10.65 [8.04, 13.26]	7.82×10^{-12}	Yes
$GABA_A$	@2.5%	Equal-weight	100	21.14	12.70	8.45 [6.17, 10.73]	2.17×10^{-10}	Yes
$GABA_A$	@5%	Boltz-2 confidence	100	14.28	10.03	4.25 [2.81, 5.68]	1.63×10^{-7}	Yes
$GABA_A$	@5%	Equal-weight	100	14.28	9.89	4.39 [3.30, 5.47]	8.93×10^{-12}	Yes
$GABA_A$	@10%	Boltz-2 confidence	100	8.35	8.75	-0.40 [-0.83, 0.03]	0.968	No
$GABA_A$	@10%	Equal-weight	100	8.35	6.79	1.56 [1.07, 2.05]	2.94×10^{-8}	Yes
$GABA_A$	@20%	Boltz-2 confidence	100	4.69	4.40	0.29 [0.09, 0.50]	0.005	Yes
$GABA_A$	@20%	Equal-weight	100	4.69	4.18	0.51 [0.31, 0.71]	4.84×10^{-6}	Yes

References

[1] Marcus Olivecrona, Thomas Blaschke, Ola Engkvist, and Hongming Chen. Molecular de novo design through deep reinforcement learning. *Journal of Cheminformatics*, 9(1):48, 2017.

[2] Anna Gaulton, Louisa J. Bellis, A. Patricia Bento, Jon Chambers, Mark Davies, Anne Hersey, Yvonne Light, Shaun McGlinchey, David Michalovich, Bissan Al-Lazikani, and John P. Overington. ChEMBL: A large-scale bioactivity database for drug discovery. *Nucleic Acids Research*, 40(D1):D1100–D1107, 2012.

[3] Rustam Zhumagambetov, Ferdinand Molnár, Vsevolod Peshkov, and Siamak Fazli. Transmol: Repurposing a language model for molecular generation. *RSC Advances*, 11(42):25921–25932, 2021.

[4] Ashish Vaswani, Noam Shazeer, Niki Parmar, Jakob Uszkoreit, Llion Jones, Aidan N. Gomez, Łukasz Kaiser, and Illia Polosukhin. Attention is all you need. In *Advances in Neural Information Processing Systems*, volume 30, pages 5998–6008, 2017.

[5] Daniil Polykovskiy, Alexander Zhebrak, Benjamin Sanchez-Lengeling, Sergey Golovanov, Oktai Tatanov, Stanislav Belyaev, Rauf Kurbanov, Aleksey Artamonov, Vladimir Aladinskiy, Mark Veselov, Artur Kadurin, Simon Johansson, Hongming Chen, Sergey Nikolenko, Alán Aspuru-Guzik, and Alex Zhavoronkov. Molecular sets (MOSES): A benchmarking platform for molecular generation models. *Frontiers in Pharmacology*, 11:565644, 2020.

[6] Michael Moret, Lukas Friedrich, Francesca Grisoni, Daniel Merk, and Gisbert Schneider. Generative molecular design in low data regimes. *Nature Machine Intelligence*, 2(3):171–180, 2020.

[7] Thin Nguyen, Hang Le, Thomas P. Quinn, Thuc Nguyen, Trang D. Le, and Svetha Venkatesh. GraphDTA: Predicting drug–target binding affinity with graph neural networks. *Bioinformatics*, 37(8):1140–1147, 2021.

[8] Matthias Fey and Jan Eric Lenssen. Fast graph representation learning with PyTorch geometric. *arXiv preprint arXiv:1903.02428*, 2019.

[9] Michael K. Gilson, Tiqing Liu, Michael Baitaluk, George Nicola, Linda Hwang, and Jenny Chong. BindingDB in 2015: A public database for medicinal chemistry, computational chemistry and systems pharmacology. *Nucleic Acids Research*, 44(D1):D1045–D1053, 2016.

[10] RDKit. RDKit: Open-source cheminformatics. https://www.rdkit.org, 2023. Version 2023.09.1.

[11] Oleg Trott and Arthur J. Olson. AutoDock Vina: Improving the speed and accuracy of docking with a new scoring function, efficient optimization, and multithreading. *Journal of Computational Chemistry*, 31(2):455–461, 2010.

[12] Jerome Eberhardt, Diogo Santos-Martins, Andreas F. Tillack, and Stefano Forli. AutoDock Vina 1.2.0: New docking methods, expanded force field, and Python bindings. *Journal of Chemical Information and Modeling*, 61(8):3891–3898, 2021.

[13] Thomas A. Halgren. Merck Molecular Force Field. I. basis, form, scope, parameterization, and performance of MMFF94. *Journal of Computational Chemistry*, 17(5–6):490–519, 1996.

[14] Sunghwan Kim, Jie Chen, Tiejun Cheng, Asta Gindulyte, Jia He, Siqian He, Qingliang Li, Benjamin A. Shoemaker, Paul A. Thiessen, Bo Yu, Leonid Zaslavsky, Jian Zhang, and Evan E. Bolton. PubChem 2019 update: Improved access to chemical data. *Nucleic Acids Research*, 47(D1):D1102–D1109, 2019.

[15] Teague Sterling and John J. Irwin. ZINC 15: Ligand discovery for everyone. *Journal of Chemical Information and Modeling*, 55(11):2324–2337, 2015.
